# Synthesis of *N*‐Substituted Pyrrole‐2,5‐Dicarboxylic Acids from d‐Galactaric Acid

**DOI:** 10.1002/cssc.202501106

**Published:** 2025-08-18

**Authors:** Jan‐Simon Jeshua Friedrichs, Kai Stirnweiß, Corinna Urmann, Volker Sieber

**Affiliations:** ^1^ TUM Campus Straubing for Biotechnology and Sustainability Technical University of Munich Schulgasse 16 94315 Straubing Germany; ^2^ Organic‐analytical Chemistry Weihenstephan‐Triesdorf University of Applied Sciences Schulgasse 16 94315 Straubing Germany; ^3^ Catalytic Research Center Technical University of Munich Ernst‐Otto‐Fischer‐Straße 1 85748 Garching Germany; ^4^ SynBiofoundry@TUM Technical University of Munich Schulgasse 22 94315 Straubing Germany; ^5^ School of Chemistry and Molecular Biosciences The University of Queensland 68 Copper Road St. Lucia 4072 Australia

**Keywords:** carboxylation, *N*‐alkylation, polyethylenfuranoate, pyrroles, pyrrole‐2,5‐dicarboxylic acid

## Abstract

Pyrrole‐2,5‐dicarboxylic acid (PDCA) and its *N*‐substituted derivatives are interesting building blocks for macromolecular applications. However, only a few published procedures describe the synthesis of PDCA derivatives. These procedures often suffer from low yields, the formation of unwanted side products, harsh reaction conditions, the use of toxic and expensive reagents, or the unavailability of certain derivatives. Here, a facile and scalable six‐step synthetic route is described, starting from biobased d‐galactaric acid, for the production of *N*‐alkylated and *N*‐arylated PDCAs, achieving total yields of up to 45%. In addition, the E‐factor of the presented synthesis route is decreased by a factor of 14 compared to already established methods.

## Graphical abstract

Straightforward synthesis route to *N*‐substituted pyrrole‐2,5‐dicarboxylic acids. Starting from biobased d‐galactaric acid. Up to 45% yield. R = alkyl, benzyl, aryl.

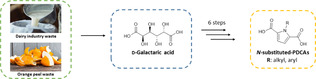

## 1 Introduction

**Figure 1:**
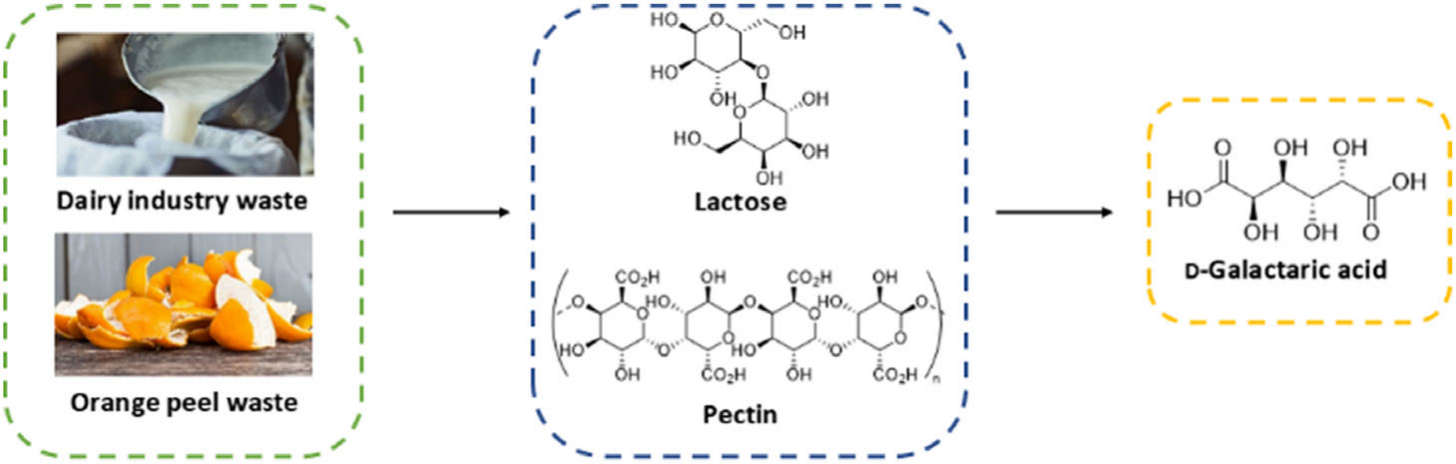
Biogenic waste streams for production of d‐galactaric acid.

Utilization of biobased starting materials to produce bulk and fine chemicals has received great attention over the past decades.^[^ ^1^ ^]^ In this context, d‐galactaric acid, also known as mucic acid, has gained increasing interest as a biobased precursor for highly valuable chemical compounds such as adipic acid or furan‐2,5‐dicarboxylic acid (FDCA), which are used in the production of nylon‐66 or polyethylenfuranoate (PEF).^[^ ^2^ ^]^ d‐Galactaric acid, an aldaric acid, occurs naturally in some fruits^[^ ^3^ ^]^ but is primarily produced on an industrial scale from the hexose d‐galactose or the disaccharide lactose through oxidation with nitric acid.^[^ ^2^, ^4^ ^]^Since d‐galactose is widely found in renewable resources, such as plants,^[^ ^5^ ^]^ polysaccharides from marine microalgae,^[^ ^6^ ^]^ or in dairy waste streams,^[^ ^2^ ^]^ it is considered a valuable biobased platform chemical. In particular, the utilization of dairy waste streams such as sweet or acid whey, which are side‐products of cheese and yogurt production, as a valuable source of d‐galactose, highlights its significance as a biogenic precursor for d‐galactaric acid production and other platform chemicals (**Figure** **1**).^[^ ^1^, ^2^, ^7^ ^]^ Besides d‐galactose, d‐galactaric acid can also be produced from d‐galacturonic acid (d‐galUA), another sugar acid derived from d‐galactose.^[^ ^8^ ^]^ d‐Galacturonic acid is a major component of the naturally occurring polymer pectin, which can be extracted from inexpensive biomass sources such as orange peel waste from the juice industry or sugar beet pulp.^[^ ^8^, ^9^ ^]^ Pectin can be hydrolyzed using pectinases,^[^ ^8^, ^10^ ^]^ and the resulting d‐galUA can subsequently be oxidized either chemically using gold‐catalysts^[^ ^11^ ^]^ or enzymatically^[^ ^8^, ^12^ ^]^ to produce d‐galactaric acid.

In this work, we describe a procedure for utilizing d‐galactaric acid in the production of *N*‐substituted pyrrole‐2,5‐dicarboxylic acids. 1*H*‐Pyrrole‐2,5‐dicarboxylic acid (PDCA) is a five‐membered heterocyclic compound that serves as an important building block in macromolecular chemistry. PDCAs have been incorporated into metal–organic frameworks (MOF) used for the separation of gas mixtures consisting of ethane/ethylene^[^ ^13^ ^]^ or ethylene/CO_2_.^[^ ^14^ ^]^ Additionally, they have been used as monomers for the production of a polyethylenfuranoate (PEF) copolymer in combination with furan‐2,5‐dicarboxylic acid (FDCA) and ethylene glycol.^[^ ^15^ ^]^ Moreover, PDCA‐strapped Calix[4]pyrroles have been employed as molecular sensors for the detection of chloride^[^ ^16^ ^]^ or azide^[^ ^17^ ^]^ anions. Cytotoxic substances derived from the naturally occurring compound CC‐1065 (*Streptomyces zelensis*)^[^ ^18^ ^]^ have also been synthesized, integrating the PDCA motif into their molecular backbone.^[^ ^19^ ^]^ Furthermore, PDCA has been reported to act as an antibiotic accelerant against *Pseudomonas aeruginosa*.^[^ ^20^ ^]^

Especially, the application of PDCAs as a component of a PEF‐copolymer shows great potential. The primary advantage of PDCA lies in its nitrogen–hydrogen bond, which can be substituted with various functional moieties. This modification can influence the properties of a PEF copolyester containing PDCA, including its crystallinity, hydrophobicity, and optical and electrostatic characteristics.^[^ ^21^ ^]^

Since there are many different applications, a facile synthesis of PDCAs on an appropriate scale, with low waste production and a high yield, is needed. While PDCA itself can be found and extracted from plants,^[^ ^22^ ^]^ or fungi,^[^ ^20^ ^]^ the synthesis of *N*‐substituted derivatives is not trivial. Recently, we published a five‐step synthetic route starting from pyrrole to synthesize various *N*‐alkylated PDCAs.^[^ ^23^ ^]^ Although this route provided good and reproducible yields, it had several drawbacks, including the use of expensive and toxic reagents such as di‐*tert*‐butyl dicarbonate (DiBoc), methyl chloroformate, and *n*‐butyllithium (n‐BuLi), as well as harsh reaction conditions. Moreover, this route is not suitable for the synthesis of *N*‐arylated PDCAs, as general nitrogen–carbon cross‐coupling conditions did not lead to the formation of the corresponding compounds.^[^ ^23^ ^]^

**Figure 2:**
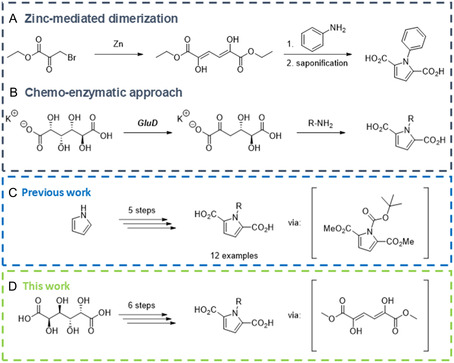
Different PDCA synthesis approaches. A) Starting from ethyl bromopyruvate.^[^ ^25^ ^]^ B) Chemo‐enzymatic approach using d‐glucaric acid as starting material.^[^ ^24^ ^]^ C) Synthesis of *N*‐alkylated PDCAs starting from pyrrol.^[^ ^23^ ^]^ D) Synthesis of *N*‐alkylated and *N*‐arylated PDCAs starting from d‐galactaric acid.

Another recently published work describes the chemo‐enzymatic synthesis of both *N*‐alkylated and *N*‐arylated PDCAs starting from d‐glucaric acid involving a Paal–Knorr cyclization.^[^ ^24^ ^]^ The corresponding PDCAs were obtained in yields of up to 20%. However, a major drawback of this route is the low selectivity of the cyclization reaction, as significant amounts of monocarboxylic acids were detected. Apart from these pathways, the most common route for PDCA synthesis proceeds via esters of 2,5‐dihydroxymuconic acid, which can also undergo cyclization in a Paal–Knorr reaction.^[^ ^25^, ^26^ ^]^ The most common method for producing this valuable intermediate is a zinc‐mediated dimerization of ethyl bromopyruvate.^[^ ^25^, ^26^ ^]^ This one‐step approach is quite convenient for synthesizing diethyl 2,5‐dihydroxymuconate but suffers from very low yields (<15%).^[^ ^25^ ^]^ Here, we describe a six‐step synthetic route for the preparation of *N*‐alkylated and *N*‐arylated PDCAs via the Paal‐Knorr educt dimethyl 2,5‐dihydroxymuconate (**Figure** **2**), achieving total PDCA yields of up to 45%.

## 2 Results and Discussion

**Scheme 1:**
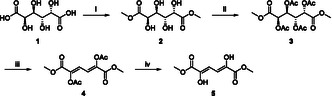
Synthesis of dimethyl 2,5‐dihydroxymuconate **5** from d‐galactaric acid **1**. i) H_2_SO_4_ (cat.), MeOH, reflux, 4d, **98%**; ii) H_2_SO_4_ (cat.), Ac_2_O, 65 °C, 3 h, **91%**; iii) DBU, CHCl_3_, 55 °C, 3.5 h, **50%**; iv) AcCl, MeOH, N_2_, 55 °C, **77%**.

The key compound for synthesizing PDCAs from d‐galactaric acid is the dimethyl 2,5‐dihydroxymuconate **5**, which is required for the Paal–Knorr reaction (**Scheme** **1**). The synthetic route presented in this study is loosely based on a procedure published by Linstead et al. in 1953.^[^ ^27^ ^]^ They described the synthesis of 2,5‐dioxoadipic acid, a tautomer of 2,5‐dihydroxymuconate, starting from d‐galactaric acid. After esterification of both carboxylic acid groups and acetylation of all free hydroxyl‐groups, they claimed to convert dimethyl 2,3,4,5‐tetra‐*O*‐acetylgalactarate to 2,5‐dioxoadipic acid in an alcoholic sodium hydroxide solution.

However, they did not provide details on the isolation or yield of the corresponding product. Instead, they used absorption spectra and the specific degradation of the product to succinic acid as structural proof. Since we were unable to reproduce their results in our laboratory, we attempted to utilize the reaction concept as a starting point for our synthetic route. The synthesis published by Linstead et al. included three independent reactions under the same reaction condition: the elimination of two equivalents of acetic acid, deacetylation of the remaining acetyl‐groups, and saponification of the methyl esters. In our approach, which also proceeds via dimethyl 2,3,4,5‐tetra‐*O*‐acetylgalactarate **3**, we decided to separate the reaction conditions required for elimination from those required for deacetylation and saponification. The esterification ^[^ ^28^ ^]^ and acetylation^[^ ^29^ ^]^ of d‐galactaric acid **1** are both well described in the literature and were performed with excellent yields of 98% and 91%, respectively (Scheme 1). To obtain pure dimethyl ester **2** or the tetra‐acetylated compound **3**, it was sufficient to wash the crude materials in hot methanol (compound **2**) and ethanol (compound **3**), respectively. For the synthesis of compound **4**, we applied conditions based on a published method for an acetic acid (HOAc) elimination using 1,8‐diazabicyclo[5.4.0]undec‐7‐ene (DBU) on peptides.^[^ ^30^ ^]^ However, the reaction conditions of the elimination reaction had to be adjusted to drive the conversion from compound **3** to compound **4**. While the reported HOAc elimination on peptides involved a temperature gradient from −15 °C to ambient temperature, the conversion from compound **3** to **4** required raising the temperature to 55 °C. In addition, we examined the effects of solvent choice, DBU equivalents, and reactant concentration. The highest yield was obtained using a concentration of 0.5–1.0 m of compound **3** with 2.2 equivalents of DBU in chloroform. Other solvents, such as tetrahydrofuran (THF) or toluene, gave similar results, but especially in the case of THF, the aqueous work‐up was more tedious, due to its intrinsic solubility in aqueous solutions. In ethanol or methanol solution, no product formation was observed. Pure compound **4** was obtained in 50% yield by recrystallizing the crude material in methanol. We also tested whether other non‐nucleophilic bases, such as potassium *tert*‐butoxide or diisopropylethylamine (DIPEA), could replace DBU, but neither resulted in conversion to the desired compound **4**.

The synthesis route continued with the deacetylation of compound **4** to dimethyl 2,5‐dihydroxymuconate **5**. In sugar chemistry, deacetylation is typically carried out using a catalytic amount of a base, such as potassium carbonate, in methanol. This reaction is known as the Zemplén reaction.^[^ ^31^ ^]^ However, these basic conditions did not lead to the deacetylation of compound **4** to **5**. Because of this, acidic conditions were tested. According to a published procedure, a catalytic amount of acetyl chloride in anhydrous methanol under a nitrogen atmosphere was used to convert compound **4** to dimethyl 2,5‐dihydroxymuconate **5**. The addition of acetyl chloride to methanol was suggested to result in the in situ formation of hydrogen chloride, which acted as the active reagent for the deacetylation of compound **4**.^[^ ^32^ ^]^ After cleavage of both acetyl groups, compound **5** precipitated from the reaction mixture and could be obtained by filtration in 77% yield. The overall yield from compounds **3** to **5** over two steps increased from 39 to 53% when the crude material from HOAc elimination is directly used for deacetylation, eliminating the need for an additional purification step.

**Figure 3:**
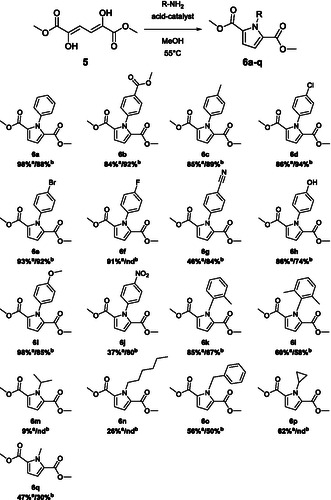
Paal–Knorr reaction. The reactions were performed using 0.495 mmol dimethyl 2,5‐dihydroxymuconate, 0.595 mmol of the corresponding amine or aniline‐derivative, and 0.149 mmol of Brønsted acid A (saccharin) or B (*p*‐TSA) in 1.00 mL methanol. Nd = not determined. ^a)^Yield for saccharin. ^b)^Yield for *p*‐TSA.

**Figure 4:**
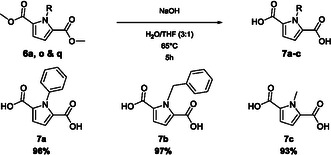
Saponification of dimethylester **6a**, **6o**, and **6q** to the corresponding dicarboxylic acids **7a**, **7b**, and **7c**.

With compound **5** in hand, the Paal–Knorr reaction could be performed using various commercially available amines and aniline derivatives. Several published procedures exist for carrying out a Paal–Knorr reaction. One method, starting from diethyl 2,5‐dihydroxymuconate, employs aniline in refluxing glacial acetic acid for cyclization to the corresponding diethyl *N*‐phenylpyrrole‐2,5‐dicarboxylate.^[^ ^25^, ^26^ ^]^ This approach was successfully reproduced starting from dimethyl 2,5‐dihydroxymuconate **5**, yielding dimethyl *N*‐phenylpyrrole‐2,5‐dicarboxylate **6a** in 77% yield. Another method utilizes a catalytic amount of saccharin as a Brønsted‐acid catalyst in methanol at 55 °C for the cyclization (**Figure** **3**). Under these conditions, compound **6a** was obtained in 98% yield. Besides saccharin, *p*‐toluene sulfonic acid (*p*‐TSA) was also tested as a catalyst, providing **6a** in 88% yield. For the Paal–Knorr reaction, 12 different aniline derivatives as well as 5 different alkyl‐ and benzylamines were screened in small‐scale reactions. Anilines substituted with electron‐withdrawing (EWG) groups tended to give higher yields when using *p*‐TSA instead of saccharin. Especially for the compounds **6g** and **6j**, bearing a cyano‐ and nitro‐group, respectively, the yields were doubled by using *p*‐TSA. Conversely, for anilines substituted with electron‐donation groups (EDG), better yields were observed when using saccharin as catalyst. Regarding alkyl amines and benzylamine, saccharin also performed better, but the overall yields were significantly lower than for the aniline derivatives. For compounds **6a**, **6o**, and **6q**, the reaction was also scaled up to produce multigram quantities of the corresponding PDCA dimethylester (11.4 g of **6a**, 89% yield; 10.9 g of **6o**, 46% yield; 6.10 g of **6q**, 37% yield). All three dimethylesters were subsequently saponified to produce the corresponding dicarboxylic acid, following the method previously described for the saponification of dimethylesters of *N*‐alkylated PDCAs (**Figure** **4**).^[^ ^23^ ^]^

The saponification was performed in excellent yields for all tested dimethylesters. For purification, a simple acid–base extraction was sufficient to obtain the dicarboxylic acids in high purity.

### 2.1 E‐Factor Comparison

To evaluate the impact and efficiency of our presented PDCA synthesis route, it is helpful to compare not only the overall yields but also the amount of waste generated throughout the process. For this purpose, the route presented in this study was compared to the alternative pathway reported by Lin et al., which is based on ethyl bromopyruvate **8**. An effective way to assess waste production in a chemical (multistep) synthesis is by using complementary metrics, such as the E‐factor.^[^ ^33^ ^]^ The E‐factor for a specific reaction is calculated by subtracting the mass of the product from the total mass of all reagents used (including solvents, if applicable) and then dividing the result by the mass of the product (SI Equation 1, Supporting Information).^[^ ^33c^ ^]^ In a multistep synthesis, the E‐factors for all reaction steps are summed up.^[^ ^33^, ^34^ ^]^

The E‐factor for the synthesis route presented in this study is based on the amount of *N*‐phenylpyrrole‐2,5‐dicarboxylic acid **7a** as described in the experimental section. Using the reported yields for each step, the corresponding quantities of reagents and solvents were determined, and the E‐factor was calculated accordingly (**Table** **1**).

**Table 1:** Listed reagents, solvents and E‐factors for the PDCA synthesis route starting from d‐galactaric acid.

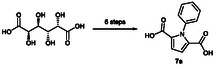				
Reaction step				
Step 6 (Saponification)	Product [g]	Reagents and Solvents	m [g]	E‐factor
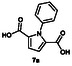	9.61	6a	11.2	17.5
		THF	44.4
		NaOH (2.5 M)	122
Step 5 (Cyclization)				
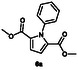	11.2	5	8.91	6.62
		Aniline	4.52
		Saccharin	2.22
		MeOH	69.6
Step 3 + 4 (Deacetylation and Elimination)				
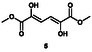	8.91	3	33.8	30.7
		DBU	27.1
		Chloroform	123
		Acetyl chloride	4.90
		MeOH	93.4
Step 2 (Acetylation)				
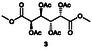	33.8	2	21.8	3.18
		Sulfuric acid	2.00
		Acetic anhydride	118
Step 1 (Esterfication)				
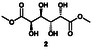	21.8	Galactaric acid	19.6	4.95
		Sulfuric acid	1.80
		MeOH	108
			E‐factor (total)	
			62.9	62.9

**Figure 5:**
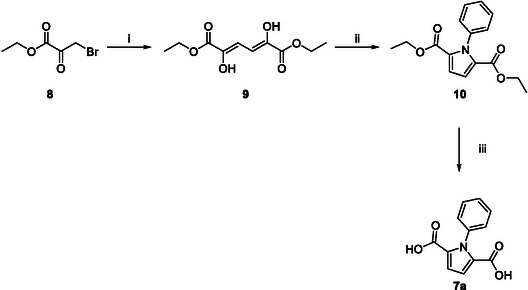
PDCA synthesis route starting from ethyl bromopyruvate **8**; i) Zn, acetone, reflux, Ar, 45 min, **11%**; ii) aniline, glacial acetic acid, reflux, Ar, 20 min, **64%**; iii) KOH, EtOH, reflux, 5 h, **76%**.^[^ ^25^ ^]^

The three‐step PDCA synthesis route described by Lin et al. starts with the zinc‐mediated dimerization of ethyl bromopyruvate **8** in refluxing acetone, yielding diethyl 2,5‐dihydroxymuconate **9** with an 11% yield. In the second step, compound **9** undergoes cyclization via a Paal–Knorr reaction in refluxing glacial acetic acid, forming diethyl *N*‐phenylpyrrole‐2,5‐dicarboxylate **10** with a 64% yield. The final step involves the saponification of compound **10** using potassium hydroxide in ethanol, producing *N*‐phenylpyrrole‐2,5‐dicarboxylic acid **7a** with a yield of 76% (**Figure** **5**).^[^ ^25^ ^]^ According to the reported yields per step, the respective amounts of reagents and solvents as well as the E‐factor were calculated and listed (Table S2, Supporting Information).

A direct comparison of the two synthesis routes reveals a significant reduction in the E‐factor, by approximately a factor of 14, when starting from d‐galactaric acid. To produce 1.00 kg of *N*‐phenylpyrrole‐2,5‐dicarboxylic acid **7a**, the d‐galactaric acid‐based route generates ≈62.9 kg of waste, whereas the route starting from ethyl bromopyruvate **8** produces around 865 kg of waste. Despite involving six steps, the route presented in this study benefits from higher yields and, more importantly, from optimized and reduced use of solvents and reagents, leading to a markedly lower E‐factor. Especially the esterification, acetylation and Paal–Knorr reaction show low E‐factors of 4.95, 3.18, and 6.62, respectively. The overall yield of **7a** from ethyl bromopyruvate **8** is ≈5% over three steps, while the route starting from d‐galactaric acid achieves a total yield of around 45%. This strong contrast is also reflected in the quantity of starting materials required. To obtain 9.61 g of **7a**, only 19.6 g of d‐galactaric acid is needed, compared to 130 g of ethyl bromopyruvate **8**. With an E‐factor of 62.9, **7a** produced from d‐galactaric acid is comparable to other monomers produced from biobased precursors. Allais et al. presented a study about the “greenness” aspect of 175 key monomers from lignin sources. According to their specific synthesis, they presented monomers with E‐factors up to 147, while most of the compounds were located at an E‐factor ranging from 0.50 to 50.0.^[^ ^34^ ^]^

However, it is necessary to mention that the presented route starting from d‐galactaric acid was optimized for larger‐scale production while the route published by Lin et al. was only performed on a laboratory scale. In addition, the amounts of solvent and reagents used for purification have been excluded as well as no recycling of solvents. The E‐factor calculated in this study is intended to highlight the green metrics of the presented route in contrast to previously established methods.

## 3 Conclusion

In this study, we presented a six‐step synthesis route to obtain *N*‐substituted PDCAs in up to 45% total yield, starting from d‐galactaric acid. This method was used to synthesize a library of 17 different *N*‐alkylated and *N*‐arylated PDCA dimethyl esters. The reaction conditions tolerated various functional groups such as esters, ether, and nitro‐, hydroxyl‐, and cyano‐substituted anilines. For three examples, the corresponding dicarboxylic acids were synthesized. Compared to a recently published route starting from pyrrole, the method presented here avoids the usage of hazardous compounds such as DiBoc, methyl chloroformate, *n*‐BuLi, or halogenated reagents, such as ethyl bromopyruvate **8**.^[^ ^23^, ^25^ ^]^ Most reactions, except for the acetylation, do not require the exclusion of oxygen or traces of water, making them accessible and reproducible even for laboratories without strong organic chemistry background. In addition, the produced amount of waste was reduced by a factor of 14 in comparison with an established route, as displayed by the corresponding E‐factors.

In addition to starting from the biobased precursor d‐galactaric acid, many of the solvents used for purification and reaction, such as methanol,^[^ ^35^ ^]^ ethanol,^[^ ^36^ ^]^ or acetic anhydride,^[^ ^37^ ^]^ can be considered green solvents, as they can be produced on industrial scale from renewable resources. To enhance both the economic and ecological value of the synthesis route, expensive and solvent‐intensive purification methods, such as column chromatography, were avoided. For the first two steps, a facile hot‐wash procedure was sufficient to obtain compounds **2** and **3** in high purity. Both reactions were easily scalable to more than 100 g. For the elimination step leading to compound **4**, followed by deacetylation to compound **5**, virtually no purification was required, as compound **5** precipitated from the reaction mixture due to its low solubility in methanol.

However, the elimination reaction to obtain compound **4** still requires the usage of halogenated and toxic chloroform, as well as a stoichiometric amount of DBU as base. As alternative, polymer‐bound DBU could be considered, as several published methods describe its incorporation into polystyrene‐matrices,^[^ ^38^ ^]^ cellulose‐nanofibers,^[^ ^39^ ^]^ or magnetically separable nanoparticles.^[^ ^40^ ^]^ Polymer‐bound DBU could be recovered after the reaction and reused for subsequent batches.

To reduce waste generation, the E‐factors of specific reaction steps should be further improved. In particular, the deacetylation, elimination, and saponification steps exhibit E‐factor values above 15, indicating significant potential for optimization.

## 4 Experimental Section

4.1

4.1.1

All chemicals were purchased from either Sigma–Aldrich, VWR, or Fisher Scientific. Solvents were either dried and stored over molar sieves (MS4Å) under a nitrogen atmosphere or bought anhydrous. For purification via dry column vacuum chromatography (DCVC), Merck silica gel 60 (0.015–0.040 mm) was used.^[^ ^41^ ^]^ Merck silica 60 (0.040–0.063 mm) was used for flash chromatography. NMR spectra were measured on a JNM‐ECA 400 MHz spectrometer from JOEL at 25 °C with the application of standard pulse programs. The chemical shifts (*δ*) are reported in ppm with reference to tetramethylsilane or the respective deuterated solvent. Coupling constants *J* are reported in Hertz (Hz). For determining CH_2_‐signals, the DEPT135°‐technique was used. For the correct assignment of the signals, 2D NMR methods, like COSY, HSQC, and HMBC, were carried out. For LC‐HRESIMS analysis, a Shimadzu system consisting of two pumps (LC‐20AD), an autosampler (SIL‐20AC HT), a column oven (CTO‐20 A), and an IT TOF (Shimadzu) was used. Separation was performed using a reverseD‐phase column (Phenomenex Kinetex C18 2.1 × 50 mm 2.6 μm) and solvents A (water + 0.1% formic acid) and B (acetonitrile + 0.1% formic acid), with a flow of 0.4 mL min^−1^, an oven temperature of 35 °C and a gradient starting with a hold of 15% solvent B for 0.15 min, rising to 95% B within 5.35 min followed by a hold of 95% B for 2.5 min. HRESIMS was recorded in positive and negative ion mode (interface voltage = +3.50 kV and −3.0 kV). The interface was set to *T* = 230 °C and a nebulizer N_2_ stream of 1.5 L min^−1^ was used. The method included MS1 with m/z 150–800 and ion accumulation of 10 ms. IR spectra were measured on a Perkin‐Elmer FT‐IR frontier UATR spectrometer. The measuring range was covered from 4000 to 525 cm^−1^. For each spectrum, 32 scans were conducted and the background subtracted.

Compound **2** and **3** were synthesized according to published procedures.^[^ ^28^, ^29^ ^]^ The PDCA synthesis is illustrated on the example of *N*‐phenylpyrrole‐2,5‐dicarboxylic acid (**7a**). The synthesis of compounds **6b**–**6q**, **7b**, and **7c** is described in the supporting information.

#### Synthesis of Dimethyl 2,5‐Diacetoxymuconate (4)

In a 1.00 L three‐neck flask equipped with a thermometer and a reflux condenser, 40.9 g of dimethyl‐2,3,4,5‐tetra‐*O*‐acetylgalactarate **3** (1.00 eq., 101 mmol) was dissolved in 400 mL of chloroform. The solution was heated to 55 °C. Subsequently, 34.0 mL (2.26 eq., 228 mmol) of 1,8‐diazabicyclo[5.4.0]undec‐7‐ene (DBU) was added, and the reaction mixture was stirred at 55 °C for 3.5 h. After completion of the reaction, the reaction mixture was concentrated under vacuum and diluted with 500 mL of dichloromethane. The organic phase was washed with 250 mL of 1 m HCl and 250 mL of a concentrated sodium chloride solution. The aqueous phase was extracted three times with each 200 mL of dichloromethane, and the combined organic phases were dried over Na_2_SO_4_. After filtration, the solvent was removed under vacuum, and the residue was dissolved in 210 mL of hot methanol (MeOH) and precipitated again at −20 °C. The crude product was recrystallized from 90 mL MeOH. The product was obtained as a white amorphous solid with a yield of 50% (14.4 g, 50.1 mmol).

^1^H NMR (400 MHz, CHLOROFORM‐D): δ 7.05 (s, 2H), 3.81 (s, 6H), 2.30 (s, 6H). ^13^C NMR (101 MHz, CHLOROFORM‐D): δ 168.50, 161.81, 141.71, 118.84, 52.97, 20.49. IR (ATR) ν: 2957, 1761, 1722, 1629, 1432, 1371, 1299, 1233, 1187, 1085, 1010, 977, 917, 899, 836, 773, 696, 646, 592, 528 cm^−1^. HRESIMS: [M+Na]^+^ *m/z* 309.0590 (calculated for C_12_H_14_O_8_, *m/z* 309.0581)

#### Synthesis of Dimethyl 2,5‐Dihydroxymuconate (5)

In a 1.00 L three‐neck flask equipped with a thermometer and a reflux condenser, 127 g of dimethyl‐2,3,4,5‐tetra‐*O*‐acetylgalactarate **3** (1.00 eq., 313 mmol) was dissolved in 500 mL of chloroform. The solution was heated to 55 °C under stirring. Subsequently, 100 mL (2.14 eq., 669 mmol) of 1,8‐diazabicyclo[5.4.0]undec‐7‐ene (DBU) was added, and the reaction mixture was stirred at 55 °C for 3.5 h. After completion of the reaction, the mixture was concentrated under vacuum and diluted with 500 mL of dichloromethane. The organic phase was washed with 250 mL of 1 m HCl and 250 mL of a concentrated sodium chloride solution. The aqueous phase was extracted three times with 200 mL of dichloromethane, and the combined organic phases were dried over Na_2_SO_4_. After filtration, the solvent was evaporated under reduced pressure, and the residue was suspended in 315 mL of anhydrous methanol under a nitrogen atmosphere.

Subsequently, 16.8 mL (0.75 eq., 236 mmol) of acetyl chloride was added dropwise to the suspension, and the mixture was stirred at 55 °C for 3 h. During the reaction time, the starting material first dissolves, followed by the precipitation of the product after a certain time. After the reaction was finished, the precipitated solid was filtered, washed with cold methanol, and dried under vacuum. The product was obtained as an ochre‐colored, amorphous solid with a yield of 53% (33.6 g, 166 mmol).

^1^H NMR (400 MHz, DMSO‐*D*_6_) δ 9.43 (s, 2H), 6.52 (s, 2H), 3.74 (s, 6H). ^13^C NMR (101 MHz, DMSO‐*D*_6_) δ 164.25, 141.83, 106.23, 52.30. IR (ATR) ν: 3390, 3105, 2959, 1686, 1631, 1442, 1332, 1249, 1159, 1119, 969, 861, 768, 696, 601, 551 cm^−1^.

#### Synthesis of Dimethyl N‐Phenylpyrrole‐2,5‐Dicarboxylate (6a)

In a 250 mL two‐neck flask equipped with a reflux condenser, 10.0 g of dimethyl (2Z,4Z)‐2,5‐dihydroxyhexa‐2,4‐dienoate **5** (1.00 eq., 49.5 mmol) and 2.34 g of saccharin (0.25 eq., 12.8 mmol) were suspended in 80 mL of MeOH. Then, 5.00 mL of freshly distilled aniline (1.11 eq., 5.11 g, 54.9 mmol) was added, and the reaction mixture was stirred at 55 °C for 5 h. After completion of the reaction, the mixture was diluted with 400 mL of EtOAc and washed with 250 mL of 1 m HCl and 250 mL of a concentrated sodium chloride solution. The aqueous phase was extracted three times with 200 mL of EtOAc, and the combined organic phases were dried over magnesium sulfate. The drying agent was filtered off, and the solvent was removed under reduced pressure. The residue was purified via DCVC (10.0 × 5.00 cm; cyclohexane/EtOAc, 20:1 to 15:1, 10:1, 9:1, 8:1). The product was obtained as a white powder with a yield of 89% (11.4 g, 44.0 mmol).

^1^H NMR (400 MHz, CHLOROFORM‐*D*) δ 7.52–7.41 (m, 3H), 7.29–7.19 (m, 2H), 7.02 (s, 2H), 3.68 (s, 6H).^13^C NMR (101 MHz, CHLOROFORM‐*D*) *δ* 160.38, 139.35, 129.06, 128.70, 128.43, 127.61, 116.90, 51.69. IR (ATR) ν: 2956, 1732, 1721, 1596, 1526, 1498, 1436, 1424, 1358, 1276, 1233, 1188, 1162, 1145, 1071, 1052, 1014, 937, 800, 751, 695, 646, 615, 607 cm^−1^. HRESIMS: [M+H]^+^ *m/z* 260.0927 (calculated for C_14_H_13_NO_4_, *m/z* 260.0917)

#### Synthesis of N‐Phenylpyrrole‐2,5‐Dicarboxylic Acid (7a)

In a 500 mL two‐neck flask equipped with a reflux condenser, 11.2 g of dimethyl *N*‐phenylpyrrol‐2,5‐dicarboxylate **6a** (1.00 eq., 43.3 mmol) was dissolved in 50 mL of THF. Subsequently, 110 mL of a 2.5 m NaOH solution was added, and the mixture was stirred at 65 °C for 5 h. After cooling the reaction mixture to room temperature, the aqueous phase was washed with 250 mL of EtOAc. The organic phase was extracted twice with 100 mL of distilled water. To precipitate the reaction product, the aqueous phase was acidified to pH 1 using 37% HCl. The product was obtained as a white powder with a yield of 96% (9.61 g, 41.6 mmol).^[^ ^25^ ^]^

^1^H NMR (400 MHz, DMSO‐*D*_6_) δ 12.53 (s, 2H), 7.44–7.32 (m, 3H), 7.26–7.17 (m, 2H), 6.95 (s, 2H). ^13^C NMR (101 MHz, DMSO‐*D*_6_) δ 160.60, 139.38, 129.59, 127.95, 127.88, 127.80, 116.37. IR (ATR) ν: 3137, 3044, 2856, 2739, 2631, 2564, 2518, 1699, 1666, 1526, 1517, 1489, 1454, 1405, 1353, 1286, 1241, 1177, 1069, 1049, 995, 981, 965, 900, 860, 812, 782, 757, 685, 637, 624, 536 cm^−1^.HRESIMS: [M–H]^−^ *m/z* 230.0452 (calculated for C_12_H_9_NO_4_, *m/z* 230.0459).

## Conflict of Interest

The authors declare no conflict of interest.

## Supporting information

Supplementary Material

## Data Availability

The data that support the findings of this study are available in the supplementary material of this article.
