# Supplementary material for: Synthesis of N‐Substituted Pyrrole‐2,5‐Dicarboxylic Acids from d‐Galactaric Acid
Source: ChemSusChem. 2025 Aug 18;18(19):e202501106. doi: 10.1002/cssc.202501106 (PMC12487735; doi:10.1002/cssc.202501106)
Supplement: Supplementary file 1 — Supplementary Material [file CSSC-18-e202501106-s001.pdf]

Supporting Information

Contents

Procedures **1**
Synthesis of Dimethyl *N*-benzylpyrrole-2,5-dicarboxylate (6o) 1
Synthesis of Dimethyl *N*-methylpyrrole-2,5-dicarboxylate (6q) 1
Synthesis of *N*-Benzylpyrrole-2,5-dicarboxylic acid (7b) 1
Synthesis of *N*-Methylpyrrole-2,5-dicarboxylic acid (7c) 1
General procedure for small-scale Paal-Knorr reaction (Method A: Saccharin) 2
General procedure for small-scale Paal-Knorr reaction (Method B: *p*-Toluenesulfonic acid) 2
Synthesis of Dimethyl *N*-phenylpyrrole-2,5-dicarboxylate (6a) 2
Synthesis of Dimethyl *N*-4-(methoxycarbonyl)phenylpyrrole-2,5-dicarboxylate (6b) 2
Synthesis of Dimethyl *N*-(4-tolyl)pyrrole-2,5-dicarboxylate (6c) 3
Synthesis of Dimethyl *N*-(4-chlorophenyl)pyrrole-2,5-dicarboxylate (6d) 3
Synthesis of Dimethyl *N*-(4-bromophenyl)pyrrole-2,5-dicarboxylate (6e) 3
Synthesis of Dimethyl *N*-(4-fluorophenyl)pyrrole-2,5-dicarboxylate (6f) 4
Synthesis of Dimethyl *N*-(4-cyanophenyl)pyrrole-2,5-dicarboxylate (6g) 4
Synthesis of Dimethyl *N*-(4-hydroxyphenyl)pyrrole-2,5-dicarboxylate (6h) 4
Synthesis of Dimethyl *N*-(4-methoxyphenyl)pyrrole-2,5-dicarboxylate (6i) 5
Synthesis of Dimethyl *N*-(4-nitrophenyl)pyrrole-2,5-dicarboxylate (6j) 5
Synthesis of Dimethyl *N*-(2-tolyl)pyrrole-2,5-dicarboxylate (6k) 5
Synthesis of Dimethyl *N*-(2,6-dimethylphenyl)pyrrole-2,5-dicarboxylate (6l) 5
Synthesis of Dimethyl *N*-isopropylpyrrole-2,5-dicarboxylate (6m) 6
Synthesis of Dimethyl *N*-hexylpyrrole-2,5-dicarboxylate (6n) 6
Synthesis of Dimethyl *N*-benzylpyrrole-2,5-dicarboxylate (6o) 6
Synthesis of Dimethyl *N*-cyclopropylpyrrole-2,5-dicarboxylate (6p) 6
Synthesis of Dimethyl *N*-methylpyrrole-2,5-dicarboxylate (6p) 7
NMR spectra of synthesized compounds: **8**
Dimethyl 2,5-diacetoxymuconate (4) 8
Dimethyl 2,5-dihydroxymuconate (5) 9
Dimethyl *N*-phenylpyrrole-2,5-dicarboxylate (6a) 10
Dimethyl *N*-[4-(methoxycarbonyl)phenyl]pyrrole-2,5-dicarboxylate (6b) 11
Dimethyl *N*-(4-tolyl)pyrrole-2,5-dicarboxylate (6c) 12
Dimethyl *N*-(4-chlorophenyl)pyrrole-2,5-dicarboxylate (6d) 13
Dimethyl *N*-(4-bromophenyl)pyrrole-2,5-dicarboxylate (6e) 14

Dimethyl *N*-(4-fluorophenyl)pyrrole-2,5-dicarboxylate (6f) 15
Dimethyl *N*-(4-cyanophenyl)pyrrole-2,5-dicarboxylate (6g) 17
Dimethyl *N*-(4-hydroxyphenyl)pyrrole-2,5-dicarboxylate (6h) 18
Dimethyl *N*-(4-methoxyphenyl)pyrrole-2,5-dicarboxylate (6i) 19
Dimethyl *N*-(4-nitrophenyl)pyrrole-2,5-dicarboxylate (6j) 20
Dimethyl *N*-(2-tolyl)pyrrole-2,5-dicarboxylate (6k) 21
Dimethyl *N*-(2,6-dimethylphenyl)pyrrole-2,5-dicarboxylate (6l) 22
Dimethyl *N*-isopropylpyrrole-2,5-dicarboxylate (6m) 23
Dimethyl *N*-hexylpyrrole-2,5-dicarboxylate (6n) 24
Dimethyl *N*-benzylpyrrole-2,5-dicarboxylate (6o) 25
Dimethyl *N*-cycloproylpyrrole-2,5-dicarboxylate (6p) 26
Dimethyl *N*-methylpyrrole-2,5-dicarboxylate (6q) 27
N-Phenylpyrrole-2,5-dicarboxylic acid (7a) 28
N-Benzylpyrrole-2,5-dicarboxylic acid (7b) 29
N-Methylpyrrole-2,5-dicarboxylic acid (7c) 30
E-factor calculation 32
Galactaric acid route 32
Ethyl bromopyruvate route 33
Literature 34

Procedures

Synthesis of Dimethyl *N*-benzylpyrrole-2,5-dicarboxylate (6o)

In a 500 mL two-neck flask equipped with a reflux condenser, 17.5 g of dimethyl (2Z,4Z)-2,5-dihydroxyhexa-2,4-dienoate **5** (1.00 eq., 86.6 mmol) and 4.76 g of saccharin (0.30 eq., 26.0 mmol) were suspended in 150 mL of MeOH. Then, 11.4 mL of benzylamine (1.20 eq., 11.1 g, 104 mmol) was added, and the reaction mixture was stirred at 55°C for 5 hours. After completion of the reaction, the reaction mixture was diluted with 400 mL of EtOAc and washed with 250 mL of 1 M HCl and 250 mL of a concentrated sodium chloride solution. The aqueous phase was extracted three times with 200 mL of EtOAc, and the combined organic phases were dried over magnesium sulfate. The drying agent was filtered off, and the solvent was removed under reduced pressure. The residue was purified by DCVC (10.0 x 5.00 cm; cyclohexane/EtOAc, 20:1 to 15:1, 10:1, 9:1, 8:1). The product was obtained as a white, amorphous solid with a yield of 46% (10.9 g, 39.9 mmol).[1]

^{1}H NMR (400 MHz, CHLOROFORM-*D*) δ 7.27 – 7.21 (m, 2H), 7.21 – 7.15 (m, 1H), 7.03 – 6.98 (m, 2H), 6.97 (s, 2H), 6.17 (s, 2H), 3.79 (s, 6H). ^{13}C NMR (101 MHz, CHLOROFORM-*D*) δ 161.12, 138.83, 128.52, 127.77, 127.02, 126.26, 117.18, 51.75, 49.33.

Synthesis of Dimethyl *N*-methylpyrrole-2,5-dicarboxylate (6q)

In a 500 mL two-neck flask equipped with a reflux condenser, 16.9 g of dimethyl (2Z,4Z)-2,5-dihydroxyhexa-2,4-dienoate **5** (1.00 eq., 83.6 mmol), 6.75 g of methylammonium chloride (1.20 eq., 100 mmol), and 4.59 g of saccharin (0.30 eq., 25.1 mmol) were suspended in 150 mL of MeOH and stirred at 55°C for four days. Subsequently, the reaction mixture was diluted with 400 mL of EtOAc and washed with 250 mL of 1 M HCl and 250 mL of a concentrated sodium chloride solution. The aqueous phase was extracted three times with 200 mL of EtOAc, and the combined organic phases were dried over magnesium sulfate. The drying agent was filtered off, and the solvent was removed under reduced pressure. The residue was purified by DCVC (10.0 x 5.00 cm; cyclohexane/EtOAc, 10:1 to 9:1, 8:1, 7:1, 6:1). The product was obtained as a white, amorphous powder with a yield of 37% (6.10 g, 30.9 mmol).[1]

^{1}H NMR (400 MHz, CHLOROFORM-*D*) δ 6.86 (s, 2H), 4.26 (s, 3H), 3.84 (s, 6H). ^{13}C NMR (101 MHz, CHLOROFORM-*D*) δ 161.44, 127.92, 116.41, 51.69, 34.60.

Synthesis of *N*-Benzylpyrrole-2,5-dicarboxylic acid (7b)

In a 500 mL two-neck flask equipped with a reflux condenser, 10.9 g of dimethyl *N*-benzylpyrrol-2,5-dicarboxylate **6o** (1.00 eq., 39.9 mmol) was dissolved in 50 mL of THF. Subsequently, 150 mL of a 2.5 M NaOH solution was added, and the mixture was stirred at 65°C for 5 hours. After cooling the reaction mixture to room temperature, the aqueous phase was washed with 250 mL of EtOAc. The organic phase was extracted twice with 100 mL of distilled water. To precipitate the reaction product, the aqueous phase was acidified to pH 1 using 37% HCl. The product was obtained as a white powder with a yield of 97% (9.48 g, 38.7 mmol).[1]

^{1}H NMR (400 MHz, DMSO-D_6) δ 7.27 (t, *J* = 7.5 Hz, 2H), 7.22 – 7.11 (m, 1H), 6.93 (s, 2H), 6.89 (d, *J* = 7.1 Hz, 2H), 6.13 (s, 2H). ^{13}C NMR (101 MHz, DMSO-D_6) δ 162.15, 139.87, 128.88, 128.48, 127.24, 126.24, 117.20, 48.67. HRESIMS: $[M-H]^-$ m/z 244.0610 (calculated for $C_{13}H_{11}NO_4$, m/z 244.0615).

Synthesis of *N*-Methylpyrrole-2,5-dicarboxylic acid (7c)

In a 250 mL two-neck flask equipped with a reflux condenser, 5.94 g of dimethyl *N*-methylpyrrol-2,5-dicarboxylate **6q** (1.00 eq., 30.1 mmol) was dissolved in 35 mL of THF. Subsequently, 100 mL of a 2.5 M

NaOH solution was added, and the mixture was stirred at 65°C for 5 hours. After cooling the reaction mixture to room temperature, the aqueous phase was washed with 200 mL of EtOAc. The organic phase was extracted twice with 100 mL of distilled water. To precipitate the reaction product, the aqueous phase was acidified to pH 1 using 37% HCl. The product was obtained as a white powder with a yield of 93% (4.82 g, 28.5 mmol).[1]

1H NMR (400 MHz, DMSO-D6) δ 6.79 (s, 1H), 4.15 (s, 2H). 13C NMR (101 MHz, DMSO-D6) δ 162.37, 128.67, 116.31, 34.59. HRESIMS: $[M-H]^-$ *m/z* 168.0296 (calculated for $C_7H_7NO_4$, *m/z* 168.0302).

General procedure for small-scale Paal-Knorr reaction (Method A: Saccharin)

In a 1.50 mL screw-cap vial equipped with a stir bar, 100 mg dimethyl (2Z,4Z)-2,5-dihydroxyhexa-2,4-dienoate **5** (1.00 eq., 0.495 mmol) and 27.3 mg saccharin (0.30 eq., 0.149 mmol) were suspended in 1.00 mL methanol. Then, 1.20 eq. of the respective amine was added and the mixture stirred at 55°C for 3 – 24 h. Subsequently, the reaction mixture was diluted with EtOAc and washed with 1 M HCl and a saturated NaCl solution. The organic phase was dried over $MgSO_4$ before removal of the solvent under reduced pressure. The crude material was purified by column chromatography.

General procedure for small-scale Paal-Knorr reaction (Method B: *p*-Toluenesulfonic acid)

In a 1.50 mL screw-cap vial equipped with a stir bar, 100 mg dimethyl (2Z,4Z)-2,5-dihydroxyhexa-2,4-dienoate **5** (1.00 eq., 0.495 mmol) and 28.3 mg *p*-TSA (0.30 eq., 0.149 mmol) were suspended in 1.00 mL methanol. Then, 1.20 eq. of the respective amine was added and the mixture stirred at 55°C for 3 – 24 h. Subsequently, the reaction mixture was diluted with EtOAc and washed with 1 M HCl and a saturated NaCl solution. The organic phase was dried over $MgSO_4$ before removal of the solvent under reduced pressure. The crude material was purified by column chromatography.

Synthesis of Dimethyl *N*-phenylpyrrole-2,5-dicarboxylate (6a)

55.5 mg aniline (1.20 eq., 0.595 mmol) were used. For column chromatography (15.0 x 2.00 cm) a mobile phase consisting of cyclohexane/ EtOAc in a ratio of 15:1 was used.

Method A: 98% yield (125 mg, 0.483 mmol).

Method B: 88% yield (113 mg, 0.436 mmol).

^{1}H NMR (400 MHz, CHLOROFORM-*D*) δ 7.52 – 7.41 (m, 3H), 7.29 – 7.19 (m, 2H), 7.02 (s, 2H), 3.68 (s, 6H).^{13}C NMR (101 MHz, CHLOROFORM-*D*) δ 160.38, 139.35, 129.06, 128.70, 128.43, 127.61, 116.90, 51.69. IR (ATR) ν: 2956, 1732, 1721, 1596, 1526, 1498, 1436, 1424, 1358, 1276, 1233, 1188, 1162, 1145, 1071, 1052, 1014, 937, 800, 751, 695, 646, 615, 607 cm^{-1}. HRESIMS: $[M+H]^+$ *m/z* 260.0927 (calculated for $C_{14}H_{13}NO_4$, *m/z* 260.0917).

Synthesis of Dimethyl *N*-4-(methoxycarbonyl)phenylpyrrole-2,5-dicarboxylate (6b)

89.9 mg 4-Aminobenzoic acid methyl ester (1.20 eq., 0.595 mmol) were used. For column chromatography (15.0 x 2.00 cm) a mobile phase consisting of cyclohexane/ EtOAc in a ratio of 5:1 was used.

Method A: 84% yield (132 mg, 0.416 mmol).

Method B: 92% yield (144 mg, 0.455 mmol).

1H NMR (400 MHz, CHLOROFORM-D) δ 8.14 (d, J = 8.5 Hz, 2H), 7.32 (d, J = 8.6 Hz, 2H), 7.04 (s, 2H), 3.93 (s, 3H), 3.68 (s, 6H). 13C NMR (101 MHz, CHLOROFORM-D) δ 166.44, 160.26, 143.47, 130.35, 129.93, 128.99, 127.84, 117.27, 52.36, 51.80. IR (ATR) ν: 2957, 2922, 2851, 1722, 1694, 1603, 1526, 1516, 1434, 1421, 1354, 1277, 1233, 1187, 1148, 1100, 1044, 1004, 955, 939, 921, 881, 824, 807, 773, 761, 748, 704, 658, 623, 614, 542, 528 cm^{-1}. HRESIMS: $[M+H]^+$ *m/z* 318.0979 (calculated for $C_{16}H_{15}NO_6$, *m/z* 318.0972)

Synthesis of Dimethyl *N*-(4-tolyl)pyrrole-2,5-dicarboxylate (6c)

63.8 mg *p*-toluidine (1.20 eq., 0.595 mmol) were used. For column chromatography (15.0 x 2.00 cm) a mobile phase consisting of cyclohexane/ EtOAc in a ratio of 6:1 was used.

Method A: 85% yield (115 mg, 0.421 mmol).

Method B: 89% yield (120 mg, 0.441 mmol).

^{1}H NMR (400 MHz, CHLOROFORM-*D*) δ 7.26 (d, *J* = 7.8 Hz, 2H), 7.13 (d, *J* = 8.2 Hz, 2H), 7.01 (s, 2H), 3.70 (s, 6H), 2.44 (s, 3H). ^{13}C NMR (101 MHz, CHLOROFORM-*D*) δ 160.41, 138.49, 136.68, 129.19, 129.01, 127.22, 116.79, 51.70, 21.55. IR (ATR) ν: 3008, 2955, 2848, 1726, 1712, 1588, 1526, 1513, 1436, 1426, 1383, 1362, 1279, 1238, 1185, 1159, 1136, 1104, 1045, 1031, 939, 825, 805, 791, 765, 739, 657, 631, 614 cm^{-1}. HRESIMS: $[M+H]^+$ *m/z* 274.1086 (calculated for $C_{15}H_{15}NO_4$, *m/z* 274.1074).

Synthesis of Dimethyl *N*-(4-chlorophenyl)pyrrole-2,5-dicarboxylate (6d)

75.9 mg *p*-chloroaniline (1.20 eq., 0.595 mmol) were used. For column chromatography (15.0 x 2.00 cm) a mobile phase consisting of cyclohexane/ EtOAc in a ratio of 7:1 was used.

Method A: 86% yield (125 mg, 0.426 mmol).

Method B: 94% yield (137 mg, 0.465 mmol).

^{1}H NMR (400 MHz, CHLOROFORM-*D*) δ 7.42 (d, *J* = 8.7 Hz, 2H), 7.18 (d, *J* = 8.7 Hz, 2H), 7.03 (s, 2H), 3.71 (s, 6H). ^{13}C NMR (101 MHz, CHLOROFORM-*D*) δ 160.34, 137.82, 134.62, 129.03, 128.96, 128.75, 117.17, 51.81. IR (ATR) ν: 3072, 2948, 2922, 2847, 1730, 1715, 1526, 1497, 1480, 1435, 1363, 1281, 1232, 1193, 1160, 1144, 1083, 1043, 1011, 942, 837, 801, 742, 715, 654, 610, 558 cm^{-1}. HRESIMS: $[M+H]^+$ *m/z* 294.0538 (calculated for $C_{14}H_{12}NO_4Cl$, *m/z* 294.0528).

Synthesis of Dimethyl *N*-(4-bromophenyl)pyrrole-2,5-dicarboxylate (6e)

102 mg *p*-bromoaniline (1.20 eq., 0.595 mmol) were used. For column chromatography (15.0 x 2.00 cm) a mobile phase consisting of cyclohexane/ EtOAc in a ratio of 7:1 was used.

Method A: 93% yield (156 mg, 0.460 mmol).

Method B: 92% yield (154 mg, 0.455 mmol).

^{1}H NMR (400 MHz, CHLOROFORM-*D*) δ 7.57 (d, *J* = 8.5 Hz, 2H), 7.12 (d, *J* = 8.6 Hz, 2H), 7.03 (s, 2H), 3.71 (s, 6H). ^{13}C NMR (101 MHz, CHLOROFORM-*D*) δ 160.32, 138.36, 131.72, 129.29, 128.99, 122.74, 117.19, 51.81. IR (ATR) ν: 3098, 3073, 2948, 2922, 2845, 1730, 1714, 1526, 1498, 1480, 1435, 1425, 1364, 1281, 1232, 1193, 1160, 1145, 1065, 1042, 1017, 999, 941, 835, 799, 743, 725, 711, 651, 609 cm^{-1}. HRESIMS: $[M+H]^+$ *m/z* 338.0017 (calculated for $C_{14}H_{12}NO_4Br$, *m/z* 338.0022).

Synthesis of Dimethyl *N*-(4-fluorophenyl)pyrrole-2,5-dicarboxylate (6f)

In a 25 mL two-necked flask equipped with a reflux condenser, 500 mg dimethyl (2Z,4Z)-2,5-dihydroxyhexa-2,4-dienoate **5** (1.00 eq., 2.47 mmol) and 113 mg saccharin (0.25 eq., 0.618 mmol) were suspended in 5.00 mL methanol. Then, 257 µL *p*-fluoroaniline (1.10 eq., 302 mg, 2.72 mmol) were added and the mixture stirred at 55°C for 3 h. Subsequently, the reaction mixture was diluted with 100 ml EtOAc and washed with 1 M HCl and a saturated NaCl solution. The organic phase was dried over $MgSO_4$ before removal of the solvent under reduced pressure. The crude material was purified by column chromatography using a mobile phase consisting of cyclohexane/ EtOAc in a ratio of 15:1. The product was obtained as a white powder with a yield of 91% (621 mg, 2.24 mmol).

^{1}H NMR (400 MHz, CHLOROFORM-*D*) δ 7.24 – 7.18 (m, 2H), 7.17 – 7.09 (m, 2H), 7.03 (s, 2H), 3.70 (s, 6H). ^{13}C NMR (101 MHz, CHLOROFORM-*D*) δ 162.51 (d, $J^{C\text{-}F}$ = 248.1 Hz), 160.38, 135.22, 129.28 (d, $J^{C\text{-}F}$ = 8.7 Hz), 129.13, 117.07, 115.47 (d, $J^{C\text{-}F}$ = 22.9 Hz), 51.77. ^{19}F NMR (376 MHz, CHLOROFORM-*D*) δ -112.62. IR (ATR) ν: 3074, 2989, 2951, 2843, 1717, 1604, 1530, 1510, 1483, 1433, 1372, 1361, 1255, 1235, 1215, 1194, 1169, 1159, 1145, 1091, 1046, 1019, 943, 841, 821, 809, 761, 745, 661, 630, 612, 602 cm^{-1}. HRESIMS: $[M+H]^+$ *m/z* 278.0832 (calculated for $C_{14}H_{12}NO_4F$, *m/z* 278.0823).

Synthesis of Dimethyl *N*-(4-cyanophenyl)pyrrole-2,5-dicarboxylate (6g)

70.3 mg 4-aminobenzonitrile (1.20 eq., 0.595 mmol) were used. For column chromatography (15.0 x 2.00 cm) a mobile phase consisting of cyclohexane/ EtOAc in a ratio of 10:3 was used.

Method A: 46% yield (64.7 mg, 0.228 mmol).

Method B: 87% yield (122 mg, 0.431 mmol).

^{1}H NMR (400 MHz, CHLOROFORM-*D*) δ 7.75 (d, *J* = 8.3 Hz, 1H), 7.37 (d, *J* = 8.3 Hz, 1H), 7.06 (s, 1H), 3.71 (s, 3H). ^{13}C NMR (101 MHz, CHLOROFORM-*D*) δ 160.22, 143.40, 132.43, 128.94, 128.89, 118.38, 117.56, 112.76, 51.93. IR (ATR) ν: 3060, 2949, 2923, 2849, 2226, 1724, 1703, 1606, 1529, 1513, 1483, 1457, 1435, 1424, 1360, 1285, 1235, 1186, 1149, 1040, 1002, 942, 915, 844, 803, 759, 745, 661, 613, 541 cm^{-1}. HRESIMS: $[M+H]^+$ *m/z* 285.0880 (calculated for C_{15}H12N_2O_4, *m/z* 285.0880).

Synthesis of Dimethyl *N*-(4-hydroxyphenyl)pyrrole-2,5-dicarboxylate (6h)

64.9 mg *p*-hydroxyaniline (1.20 eq., 0.595 mmol) were used. For column chromatography (15.0 x 2.00 cm) a mobile phase consisting of cyclohexane/ EtOAc in a ratio of 2:1 was used.[2]

Method A: 86% yield (117 mg, 0.426 mmol).

Method B: 74% yield (101 mg, 0.366 mmol).

^{1}H NMR (400 MHz, CHLOROFORM-*D*) δ 7.06 (d, *J* = 8.7 Hz, 2H), 7.02 (s, 2H), 6.71 (d, *J* = 8.7 Hz, 2H), 6.33 (s, 1H), 3.73 (s, 6H). ^{13}C NMR (101 MHz, CHLOROFORM-*D*) δ 160.85, 156.34, 131.25, 129.12, 128.27, 117.05, 115.61, 51.89. IR (ATR) ν: 3351, 3007, 2955, 2922, 2849, 1724, 1699, 1679, 1615, 1598, 1513, 1436, 1358, 1262, 1223, 1192, 1164, 1143, 1103, 1051, 1014, 939, 919, 837, 812, 798, 751, 726, 677, 661, 642, 608 cm^{-1}. HRESIMS: $[M+H]^+$ *m/z* 276.0888 (calculated for $C_{14}H_{13}NO_5$, *m/z* 276.0866).

Synthesis of Dimethyl *N*-(4-methoxyphenyl)pyrrole-2,5-dicarboxylate (6i)

73.3 mg *p*-anisidine (1.20 eq., 0.595 mmol) were used. For column chromatography (15.0 x 2.00 cm) a mobile phase consisting of cyclohexane/ EtOAc in a ratio of 2:1 was used.

Method A: 98% yield (140 mg, 0.485 mmol).

Method B: 85% yield (122 mg, 0.421 mmol).

^{1}H NMR (400 MHz, CHLOROFORM-*D*) δ 7.16 (d, *J* = 8.7 Hz, 2H), 7.01 (s, 2H), 6.96 (d, *J* = 9.1 Hz, 2H), 3.86 (s, 3H), 3.70 (s, 6H). ^{13}C NMR (101 MHz, CHLOROFORM-*D*) δ 160.43, 159.48, 131.93, 129.12, 128.45, 116.77, 113.57, 55.40, 51.69. IR (ATR) ν: 3020, 2932, 2839, 1732, 1713, 1612, 1515, 1465, 1452, 1432, 1359, 1298, 1281, 1250, 1221, 1189, 1153, 1143, 1110, 1045, 1016, 1001, 953, 939, 912, 834, 815, 789, 758, 667, 626, 609 cm^{-1}. HRESIMS: [M+H]$^+$ *m/z* 290.1033 (calculated for $C_{15}H_{15}NO_5$, *m/z* 290.1033).

Synthesis of Dimethyl *N*-(4-nitrophenyl)pyrrole-2,5-dicarboxylate (6j)

82.2 mg *p*-nitroaniline (1.20 eq., 0.595 mmol) were used. For column chromatography (15.0 x 2.00 cm) a mobile phase consisting of cyclohexane/ EtOAc in a ratio of 5:1 was used.

Method A: 37% yield (140 mg, 0.183 mmol).

Method B: 80% yield (120 mg, 0.396 mmol).

^{1}H NMR (400 MHz, CHLOROFORM-*D*) δ 8.33 (d, *J* = 8.7 Hz, 2H), 7.42 (d, *J* = 8.7 Hz, 2H), 7.08 (s, 2H), 3.71 (s, 6H). ^{13}C NMR (101 MHz, CHLOROFORM-*D*) δ 160.25, 147.70, 145.08, 129.01, 128.98, 123.94, 117.68, 51.98. IR (ATR) ν: 3137, 3117, 3082, 3053, 3008, 2957, 2922, 2851, 1721, 1692, 1612, 1597, 1522, 1504, 1451, 1441, 1432, 1422, 1347, 1295, 1240, 1194, 1153, 1110, 1044, 1025, 999, 944, 925, 870, 857, 839, 811, 753, 728, 698, 656, 619, 607, 547, 527 cm^{-1}. HRESIMS: [M+H]$^+$ *m/z* 305.0735 (calculated for $C_{12}H_{14}N_2O_6$, *m/z* 305.0744).

Synthesis of Dimethyl *N*-(2-tolyl)pyrrole-2,5-dicarboxylate (6k)

63.8 mg *o*-toluidine (1.20 eq., 0.595 mmol) were used. For column chromatography (15.0 x 2.00 cm) a mobile phase consisting of cyclohexane/ EtOAc in a ratio of 8:1 was used.

Method A: 85% yield (115 mg, 0.421 mmol).

Method B: 67% yield (90.6 mg, 0.336 mmol).

^{1}H NMR (400 MHz, CHLOROFORM-*D*) δ 7.37 (td, *J* = 7.4, 1.4 Hz, 1H), 7.32 – 7.22 (m, 2H), 7.10 (dd, *J* = 7.7, 1.4 Hz, 1H), 7.05 (s, 2H), 3.69 (s, 6H), 1.98 (s, 3H). ^{13}C NMR (101 MHz, CHLOROFORM-*D*) δ 160.23, 138.78, 135.71, 130.15, 128.88, 128.49, 127.31, 126.12, 116.85, 51.70, 17.27. IR (ATR) ν: 2996, 2952, 2922, 1729, 1716, 1587, 1525, 1500, 1479, 1457, 1430, 1362, 1291, 1274, 1231, 1191, 1153, 1051, 1041, 1016, 1005, 941, 805, 789, 769, 752, 724, 681, 651, 529 cm^{-1}. HRESIMS: [M+H]$^+$ *m/z* 274.1092 (calculated for $C_{15}H_{15}NO_4$, *m/z* 274.1074).

Synthesis of Dimethyl *N*-(2,6-dimethylphenyl)pyrrole-2,5-dicarboxylate (6l)

72.1 mg 2,6-dimethylaniline (1.20 eq., 0.595 mmol) were used. For column chromatography (15.0 x 2.00 cm) a mobile phase consisting of cyclohexane/ EtOAc in a ratio of 7:1 was used. The product was obtained in a purity of around 90%.

Method A: 60% yield (85,3 mg, 0.297 mmol).

Method B: 58% yield (82.5 mg, 0.287 mmol).

^{1}H NMR (400 MHz, CHLOROFORM-*D*) δ 7.27 – 7.21 (m, 1H), 7.13 (d, *J* = 7.5 Hz, 2H), 7.10 – 7.05 (m, 2H), 3.70 (s, 6H), 1.90 (s, 6H). ^{13}C NMR (101 MHz, CHLOROFORM-*D*) δ 160.10, 138.15, 135.29, 128.47, 127.75, 127.65, 116.97, 51.71, 17.45. IR (ATR) ν: 2956, 2922, 2851, 1732, 1715, 1578, 1525, 1465, 1436, 1411, 1378, 1357, 1272, 1232, 1195, 1158, 1046, 1034, 1013, 938, 888, 872, 820, 802, 763, 657 cm^{-1}. HRESIMS: $[M+H]^+$ *m/z* 288.1250 (calculated for $C_{16}H_{17}NO_4$, *m/z* 288.1230).

Synthesis of Dimethyl *N*-isopropylpyrrole-2,5-dicarboxylate (6m)

35.2 mg isopropylamine (1.20 eq., 0.595 mmol) were used. For column chromatography (15.0 x 2.00 cm) a mobile phase consisting of cyclohexane/ EtOAc in a ratio of 10:1 was used.[1]

Method A: 60% yield (9.80 mg, 0.297 mmol).

Method B: not determined.

^{1}H NMR (400 MHz, CHLOROFORM-*D*) δ 6.87 (s, 2H), 5.84 (hept, *J* = 7.1 Hz, 1H), 3.84 (s, 6H), 1.60 (d, *J* = 6.9 Hz, 6H). ^{13}C NMR (101 MHz, CHLOROFORM-*D*) δ 161.53, 128.20, 117.57, 51.88, 50.16, 21.22.

Synthesis of Dimethyl *N*-hexylpyrrole-2,5-dicarboxylate (6n)

60.2 mg hexylamine (1.20 eq., 0.595 mmol) were used. For column chromatography (15.0 x 2.00 cm) a mobile phase consisting of cyclohexane/ EtOAc in a ratio of 20:1 was used.[1]

Method A: 26% yield (34.4 mg, 0.129 mmol).

Method B: not determined.

^{1}H NMR (400 MHz, CHLOROFORM-*D*) δ 6.87 (s, 2H), 4.83 – 4.75 (m, 2H), 3.84 (s, 6H), 1.78 – 1.66 (m, 2H), 1.41 – 1.21 (m, 6H), 0.93 – 0.83 (m, 3H). ^{13}C NMR (101 MHz, CHLOROFORM-*D*) δ 161.17, 127.21, 116.64, 51.67, 46.83, 31.93, 31.57, 26.53, 22.77, 14.15.

Synthesis of Dimethyl *N*-benzylpyrrole-2,5-dicarboxylate (6o)

63.8 mg benzylamine (1.20 eq., 0.595 mmol) were used. For column chromatography (15.0 x 2.00 cm) a mobile phase consisting of cyclohexane/ EtOAc in a ratio of 12:1 was used.[1]

Method A: 56% yield (75.2 mg, 0.275 mmol).

Method B: 50% yield (67.4 mg, 0.247 mmol).

^{1}H NMR (400 MHz, CHLOROFORM-*D*) δ 7.27 – 7.21 (m, 2H), 7.21 – 7.15 (m, 1H), 7.03 – 6.98 (m, 2H), 6.97 (s, 2H), 6.17 (s, 2H), 3.79 (s, 6H). ^{13}C NMR (101 MHz, CHLOROFORM-*D*) δ 161.12, 138.83, 128.52, 127.77, 127.02, 126.26, 117.18, 51.75, 49.33.

Synthesis of Dimethyl *N*-cyclopropylpyrrole-2,5-dicarboxylate (6p)

34.0 mg cyclopropylamine (1.20 eq., 0.595 mmol) were used. For column chromatography (15.0 x 2.00 cm) a mobile phase consisting of cyclohexane/ EtOAc in a ratio of 5:1 was used.[1]

Method A: 62% yield (68.6 mg, 0.307 mmol).

Method B: not determined.

^{1}H NMR (400 MHz, CHLOROFORM-*D*) δ 6.75 (s, 2H), 3.85 (s, 6H), 3.63 – 3.51 (m, 1H), 1.22 – 1.05 (m, 2H), 0.86 – 0.55 (m, 2H). ^{13}C NMR (101 MHz, CHLOROFORM-*D*) δ 160.82, 130.10, 116.10, 51.80, 30.49, 10.76. IR (ATR) ν: 3125, 3018, 2959, 2847, 1715, 1527, 1518, 1459, 1437, 1426, 1372, 1363, 1338, 1232, 1192, 1174, 1161, 1140, 1054, 1037, 1031, 1017, 939, 911, 887, 825, 803, 777, 751, 654, 632, 599, 563 cm^{-1}.

Synthesis of Dimethyl *N*-methylpyrrole-2,5-dicarboxylate (6p)

40.2 mg methyl ammonium chloride (1.20 eq., 0.595 mmol) were used. For column chromatography (15.0 x 2.00 cm) a mobile phase consisting of cyclohexane/ EtOAc in a ratio of 12:1 was used.[1]

Method A: 47% yield (46.2 mg, 0.234 mmol).

Method B: 30% yield (29.2 mg, 0.148 mmol).

^{1}H NMR (400 MHz, CHLOROFORM-*D*) δ 6.86 (s, 2H), 4.26 (s, 3H), 3.84 (s, 6H). ^{13}C NMR (101 MHz, CHLOROFORM-*D*) δ 161.44, 127.92, 116.41, 51.69, 34.60.

NMR spectra of synthesized compounds:

Dimethyl 2,5-diacetoxymuconate (4)

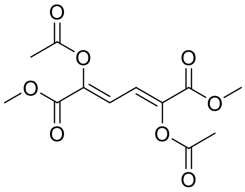

4

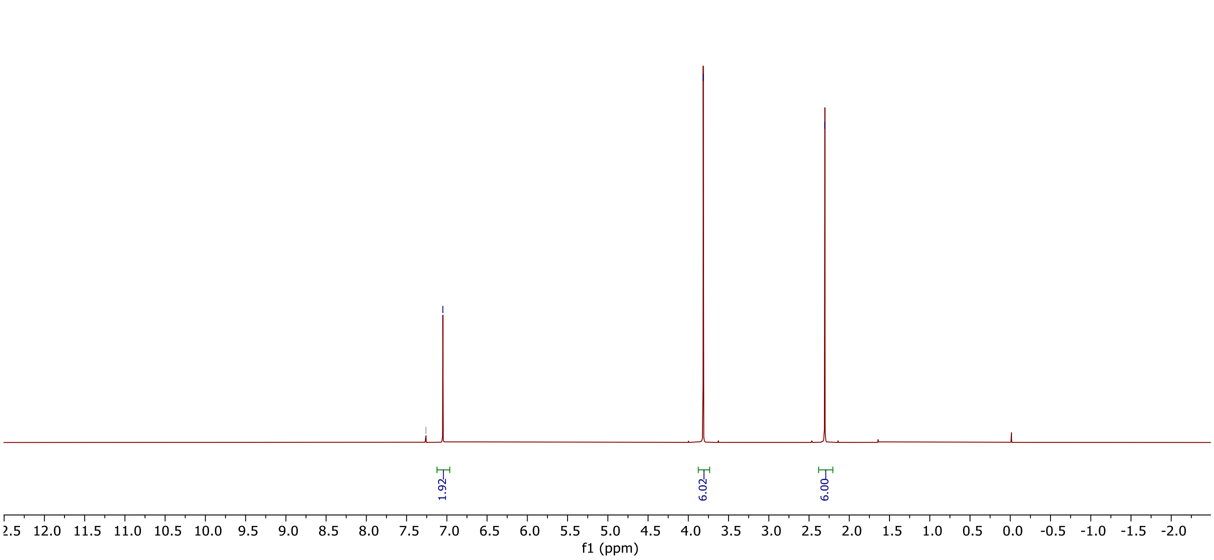

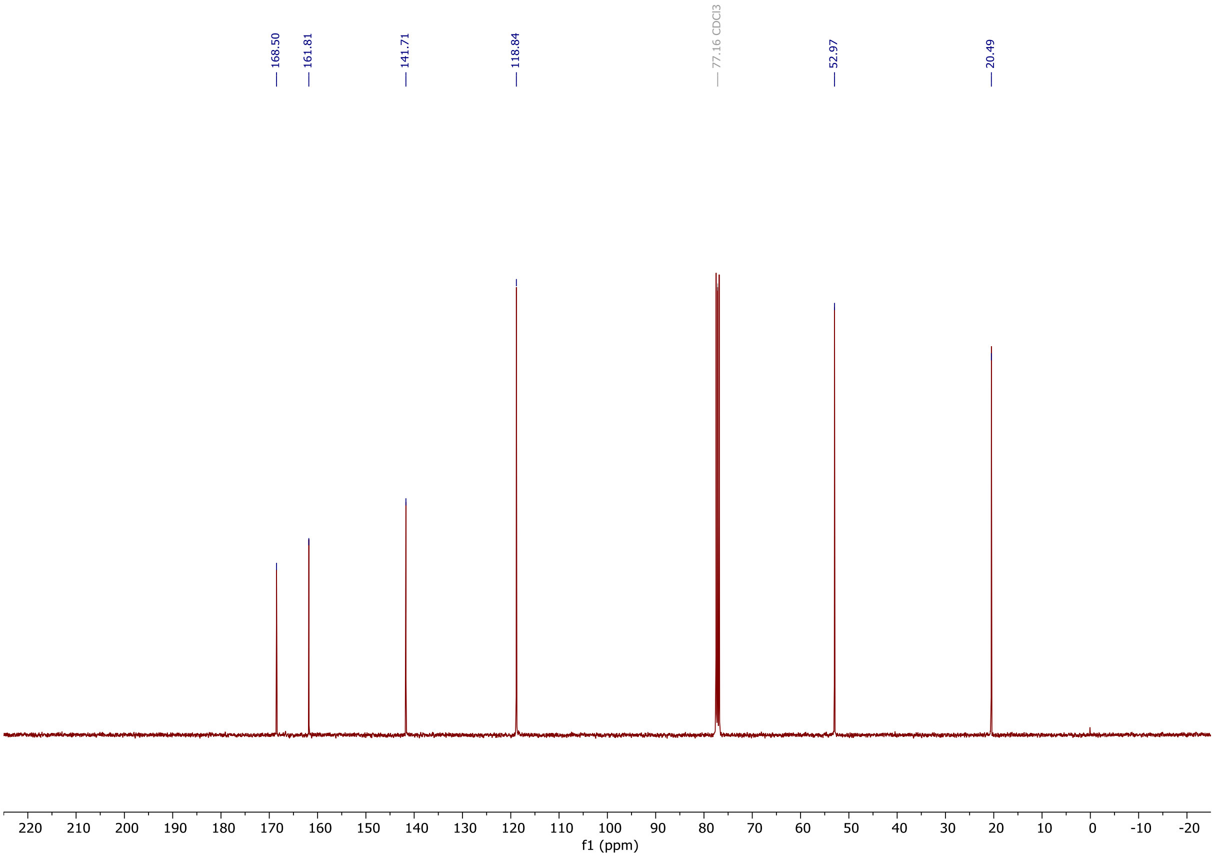

Dimethyl 2,5-dihydroxymuconate (5)

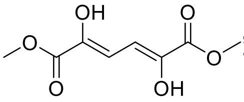

5

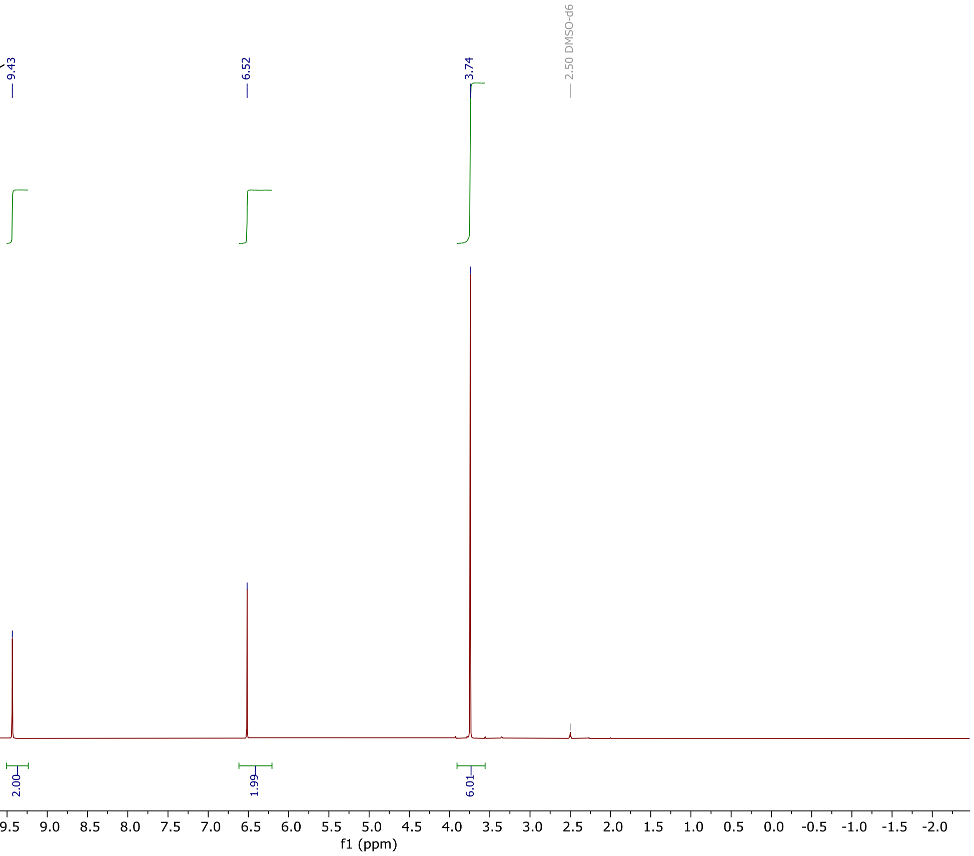

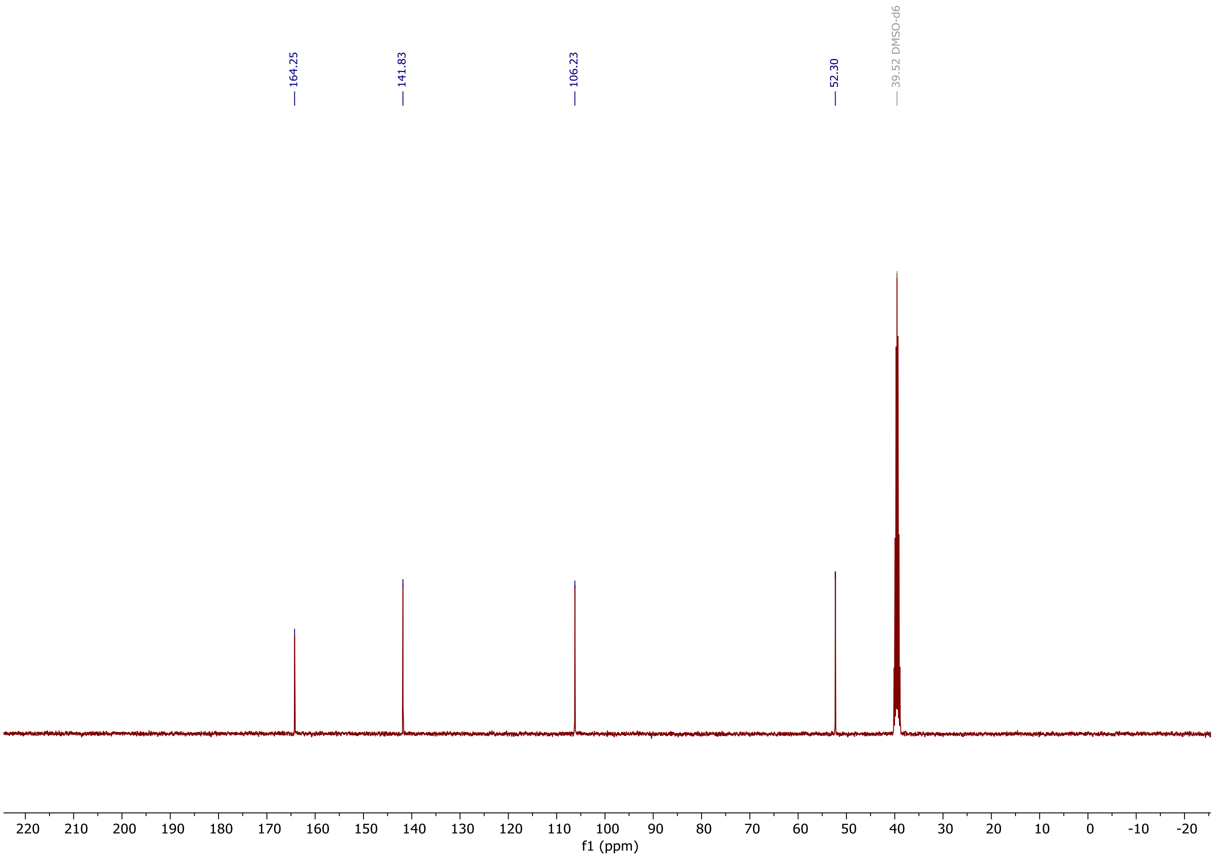

Dimethyl *N*-phenylpyrrole-2,5-dicarboxylate (6a)

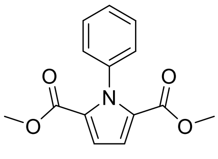

6a

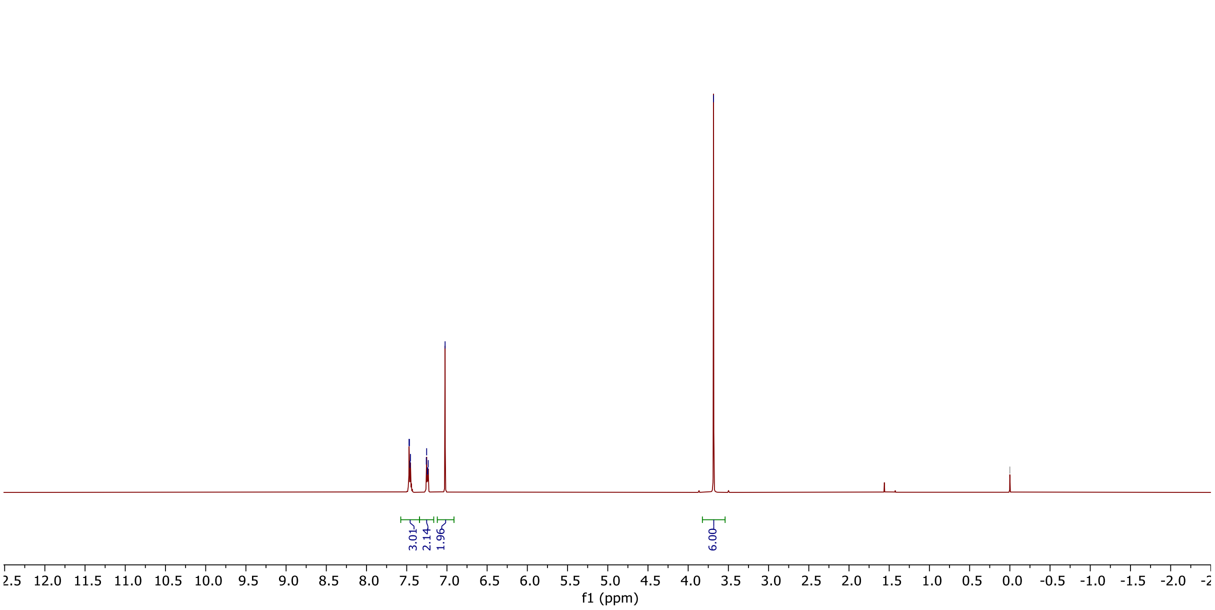

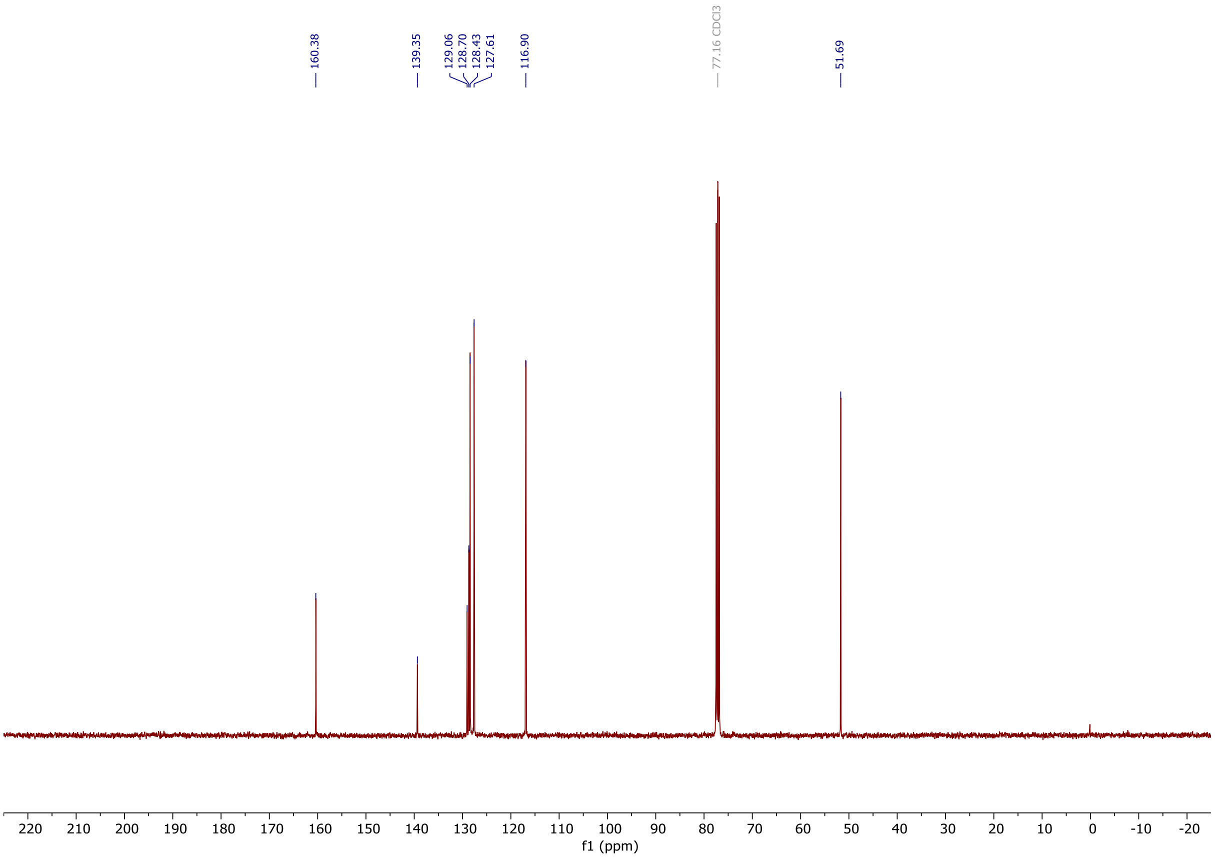

Dimethyl *N*-[4-(methoxycarbonyl)phenyl]pyrrole-2,5-dicarboxylate (6b)

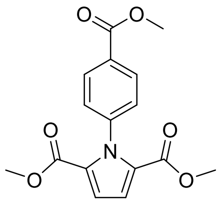

6b

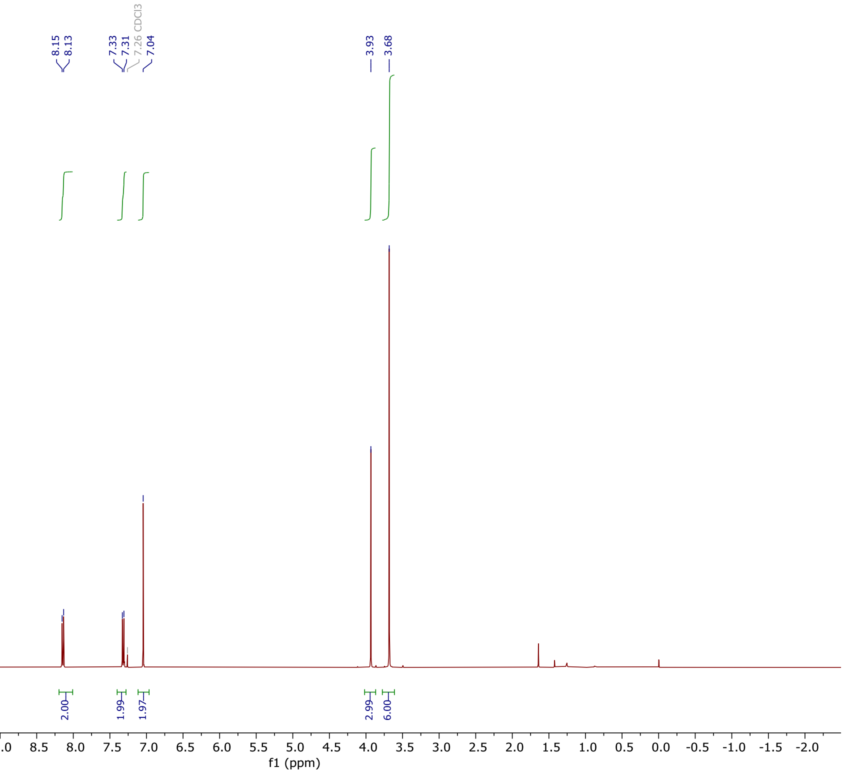

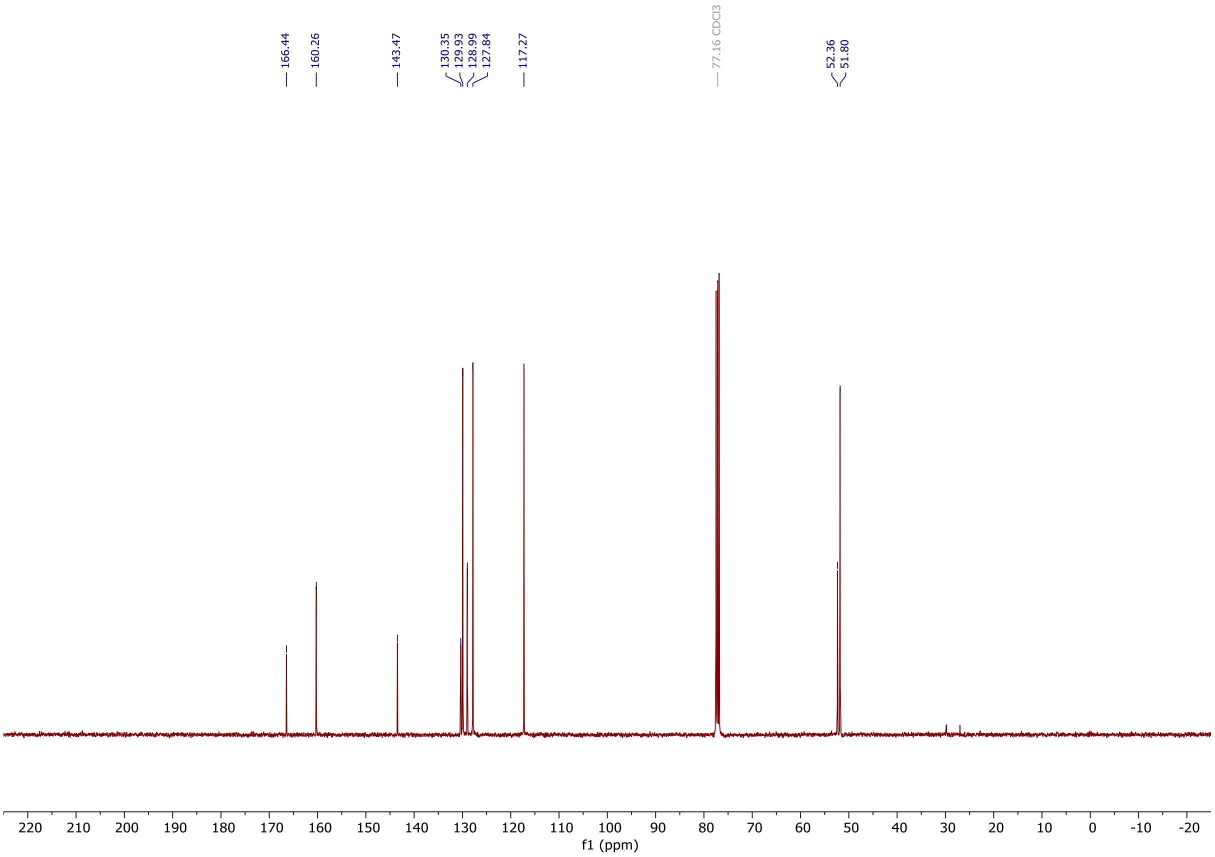

Dimethyl *N*-(4-tolyl)pyrrole-2,5-dicarboxylate (6c)

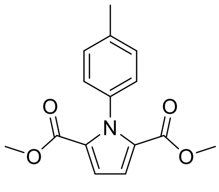

6c

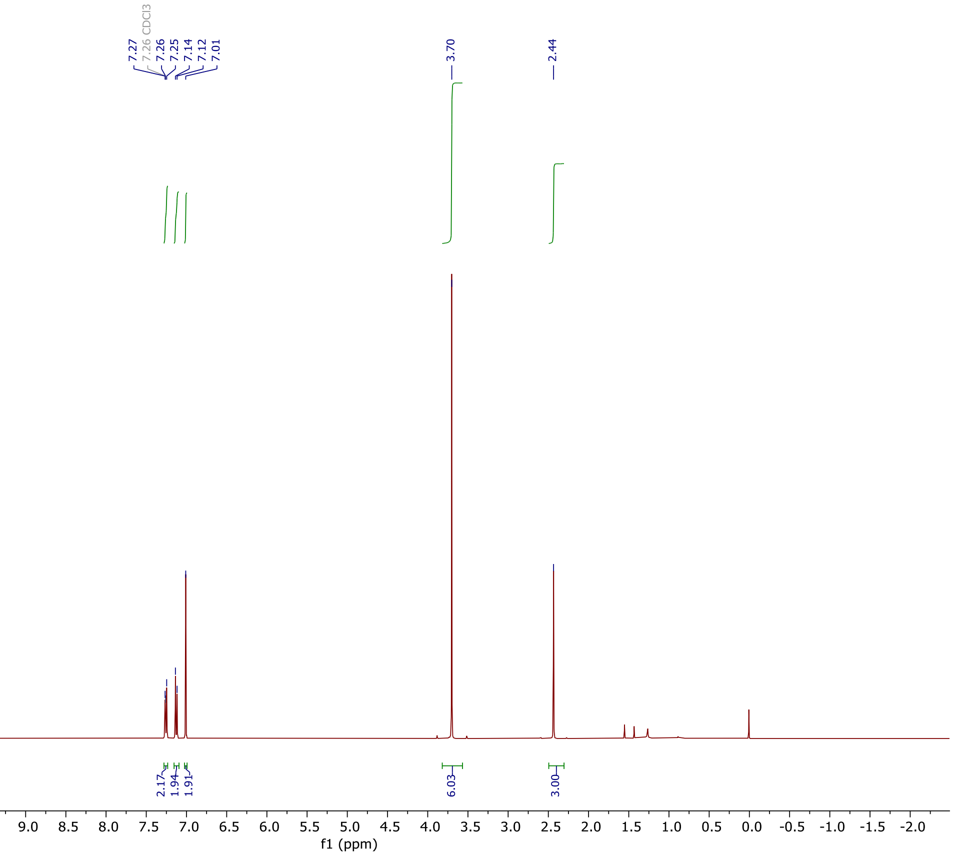

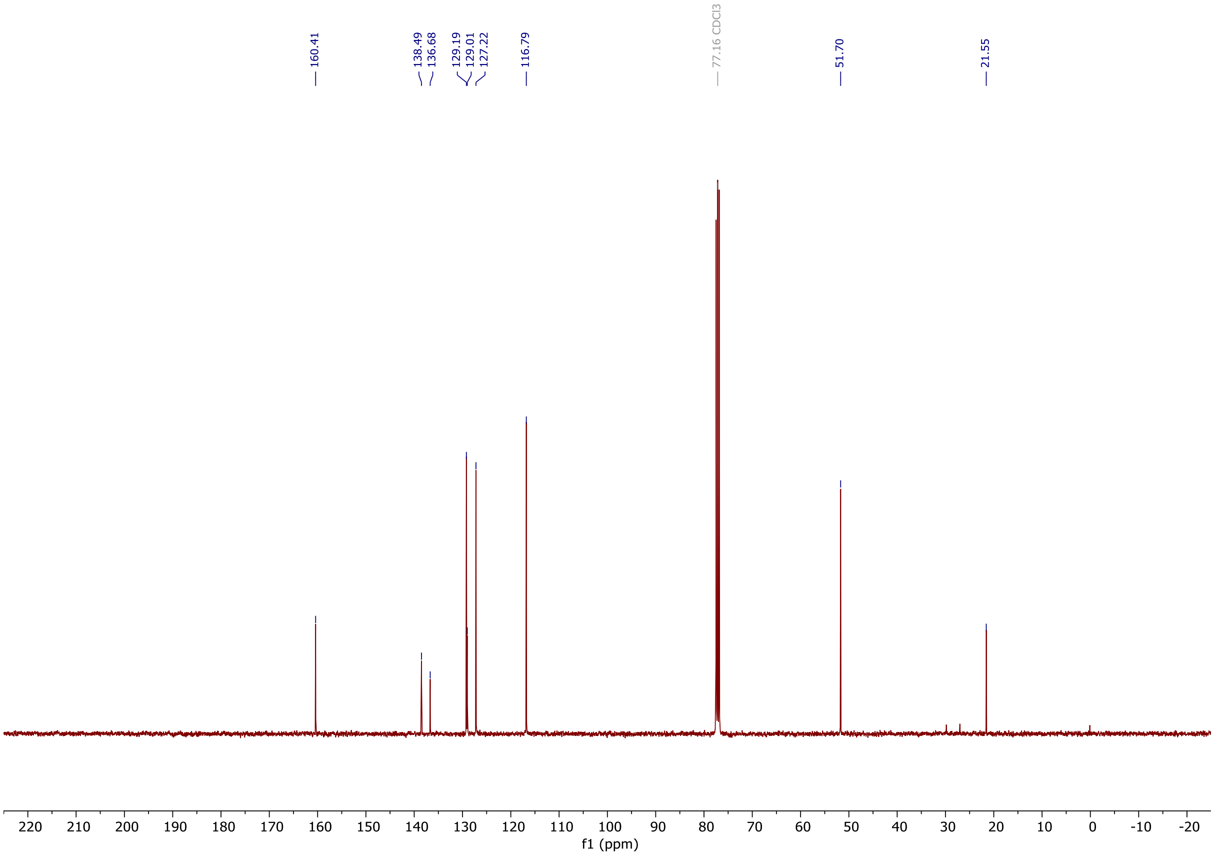

Dimethyl *N*-(4-chlorophenyl)pyrrole-2,5-dicarboxylate (6d)

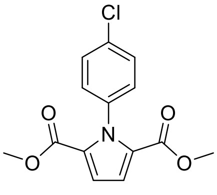

6d

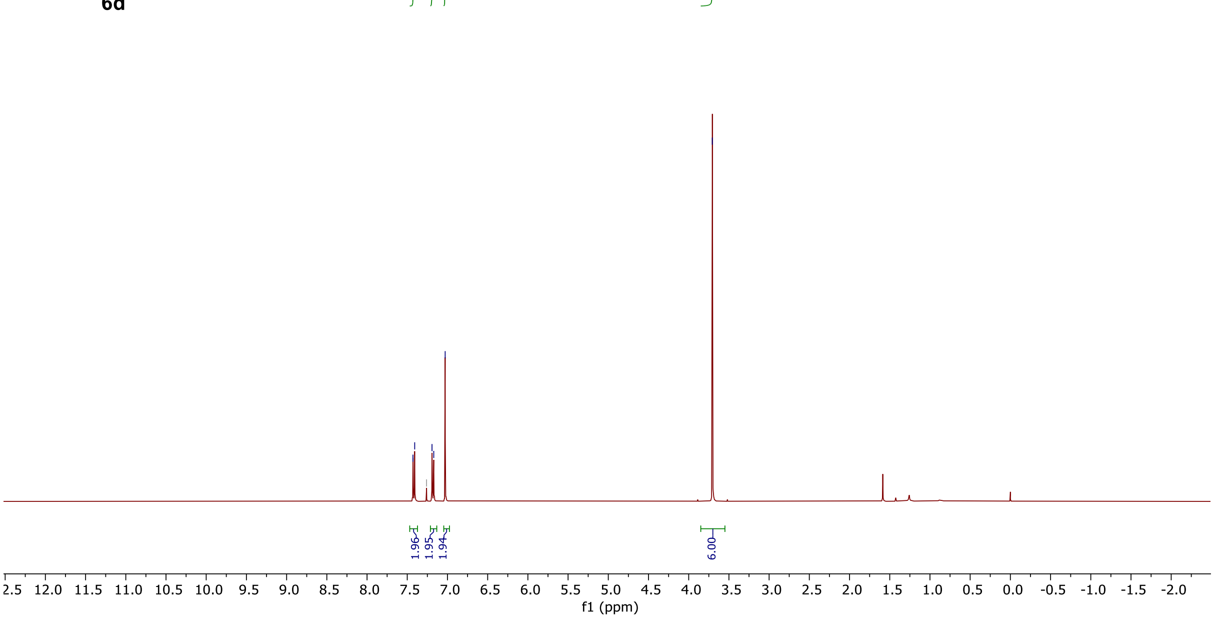

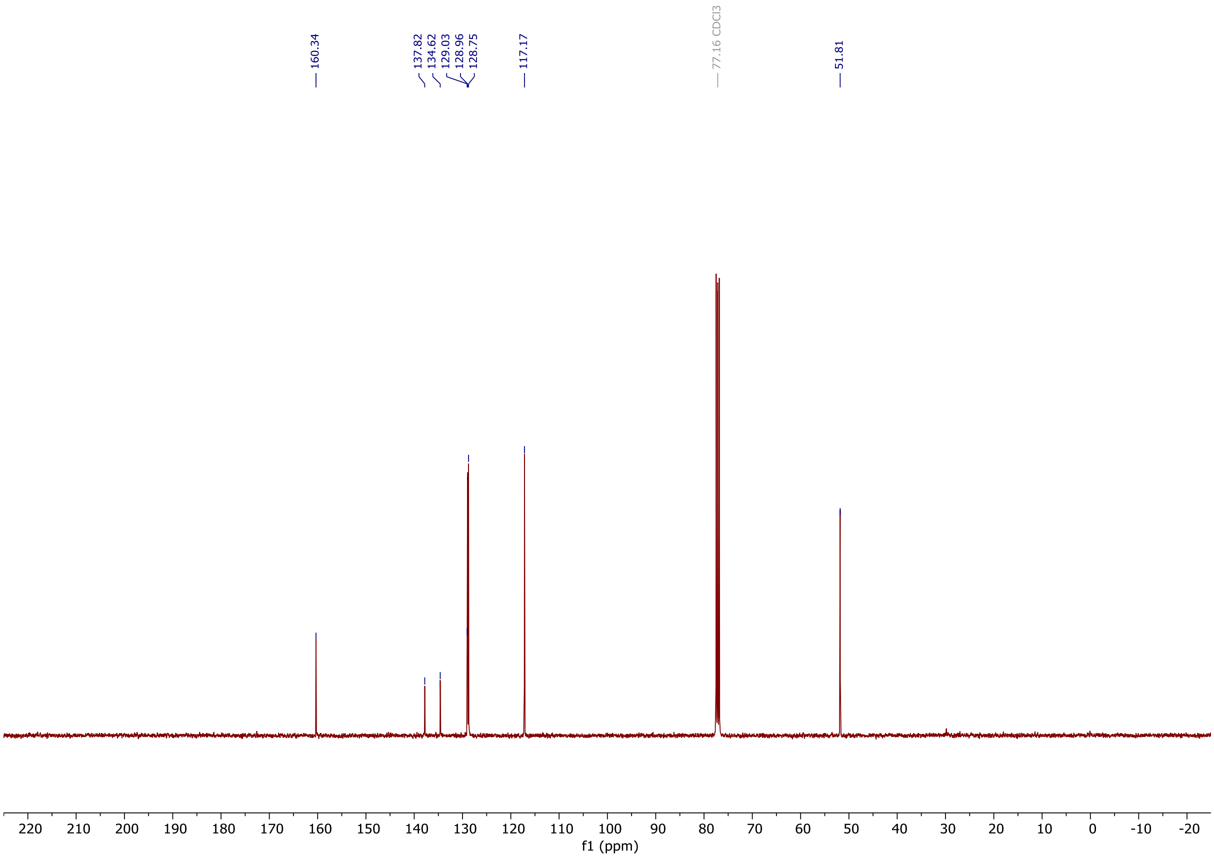

Dimethyl *N*-(4-bromophenyl)pyrrole-2,5-dicarboxylate (6e)

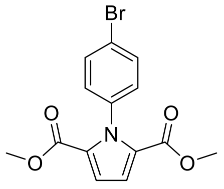

6e

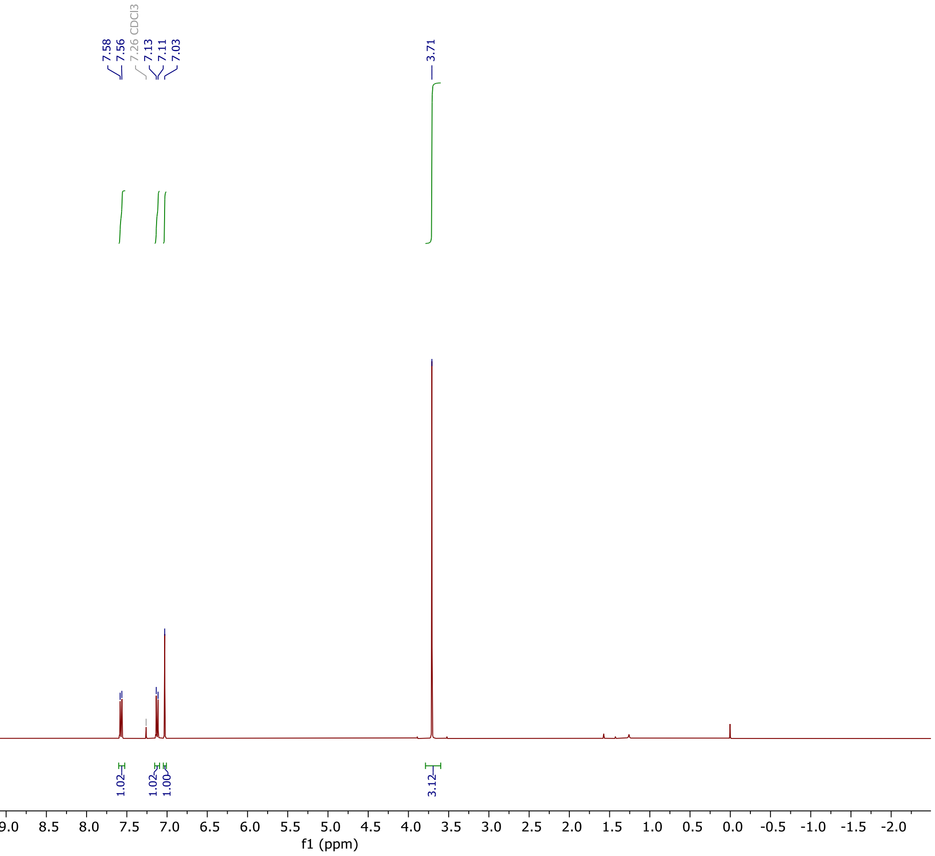

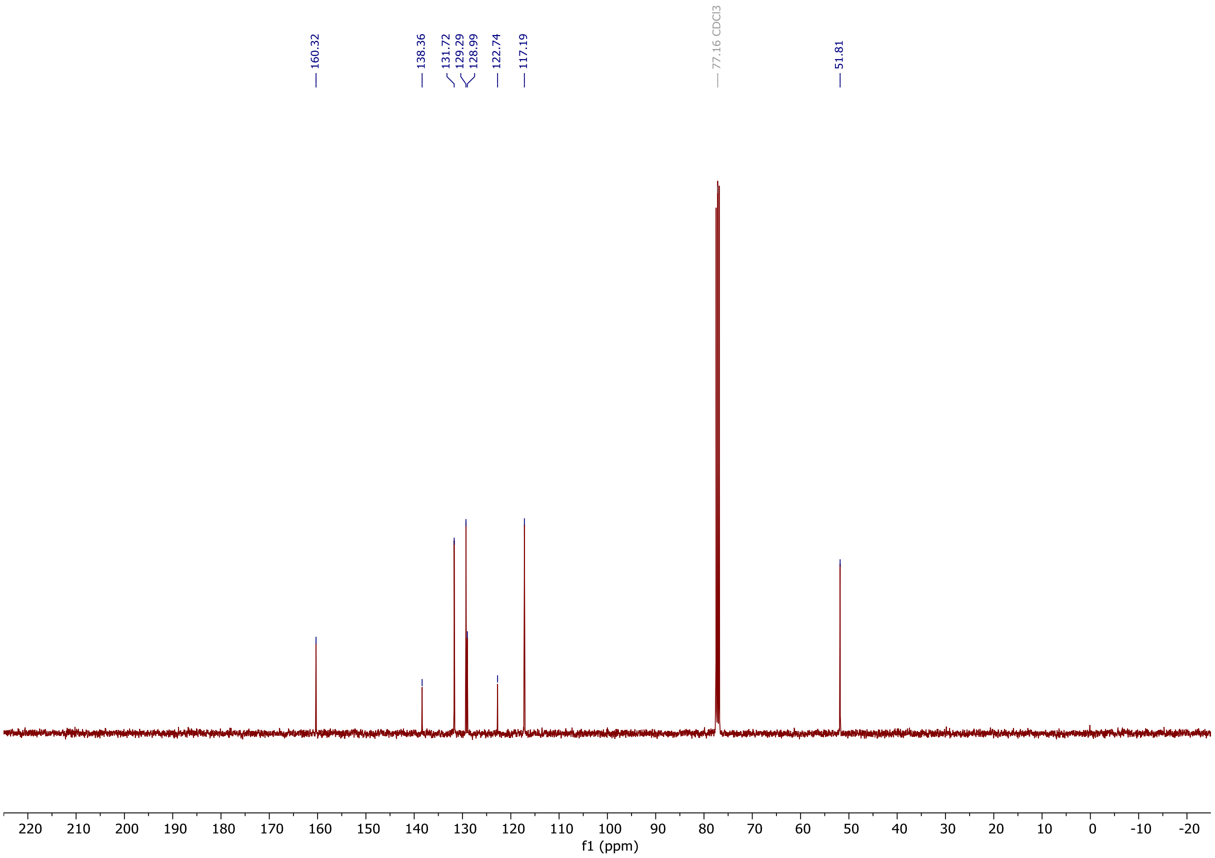

Dimethyl *N*-(4-fluorophenyl)pyrrole-2,5-dicarboxylate (6f)

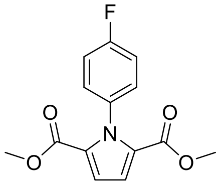

6f

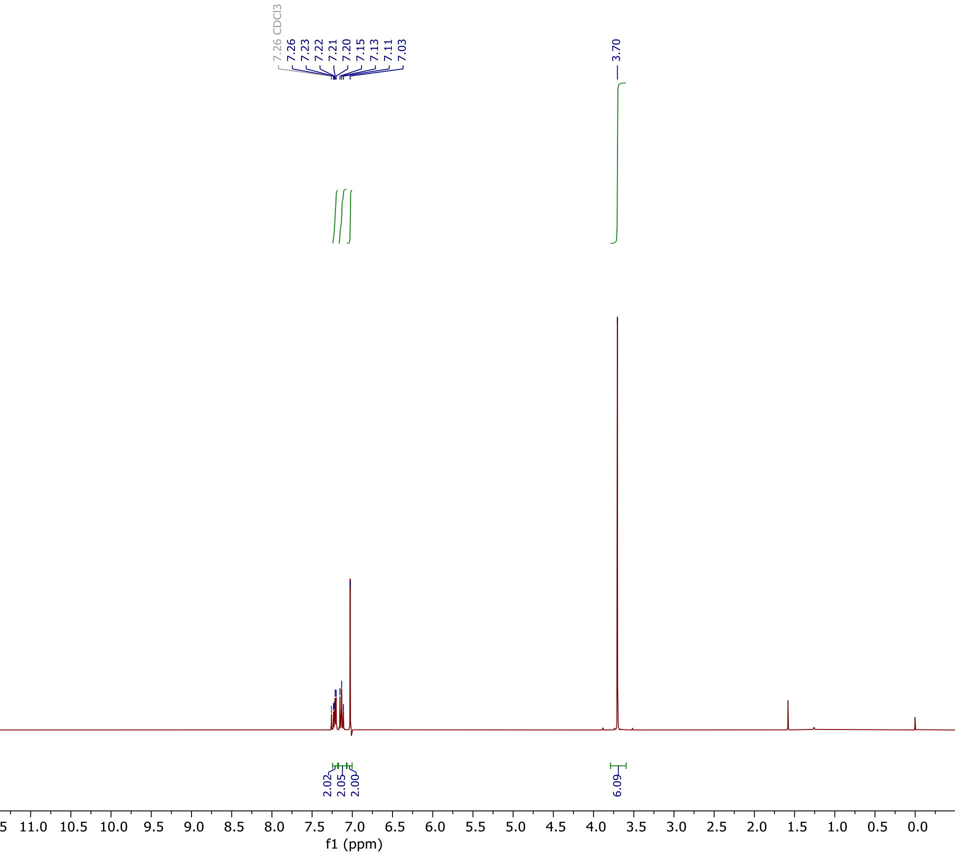

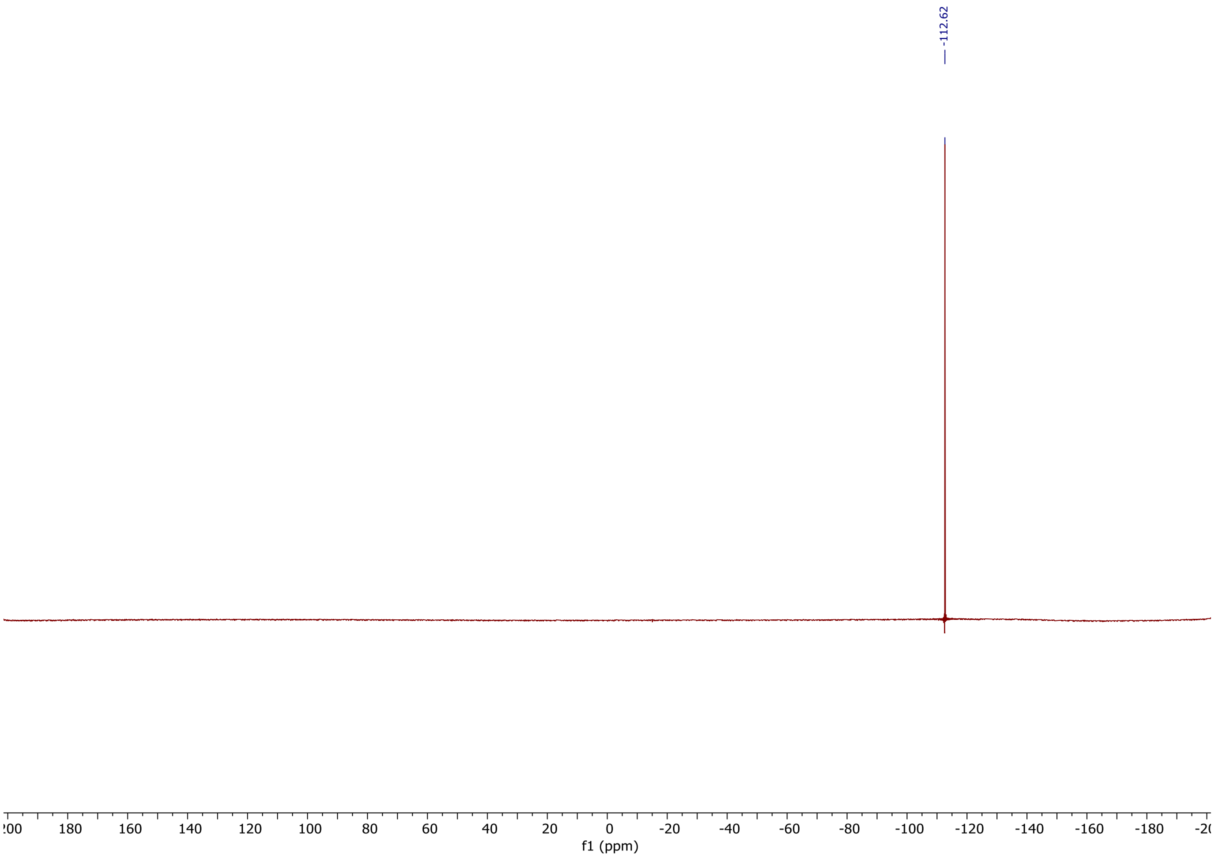

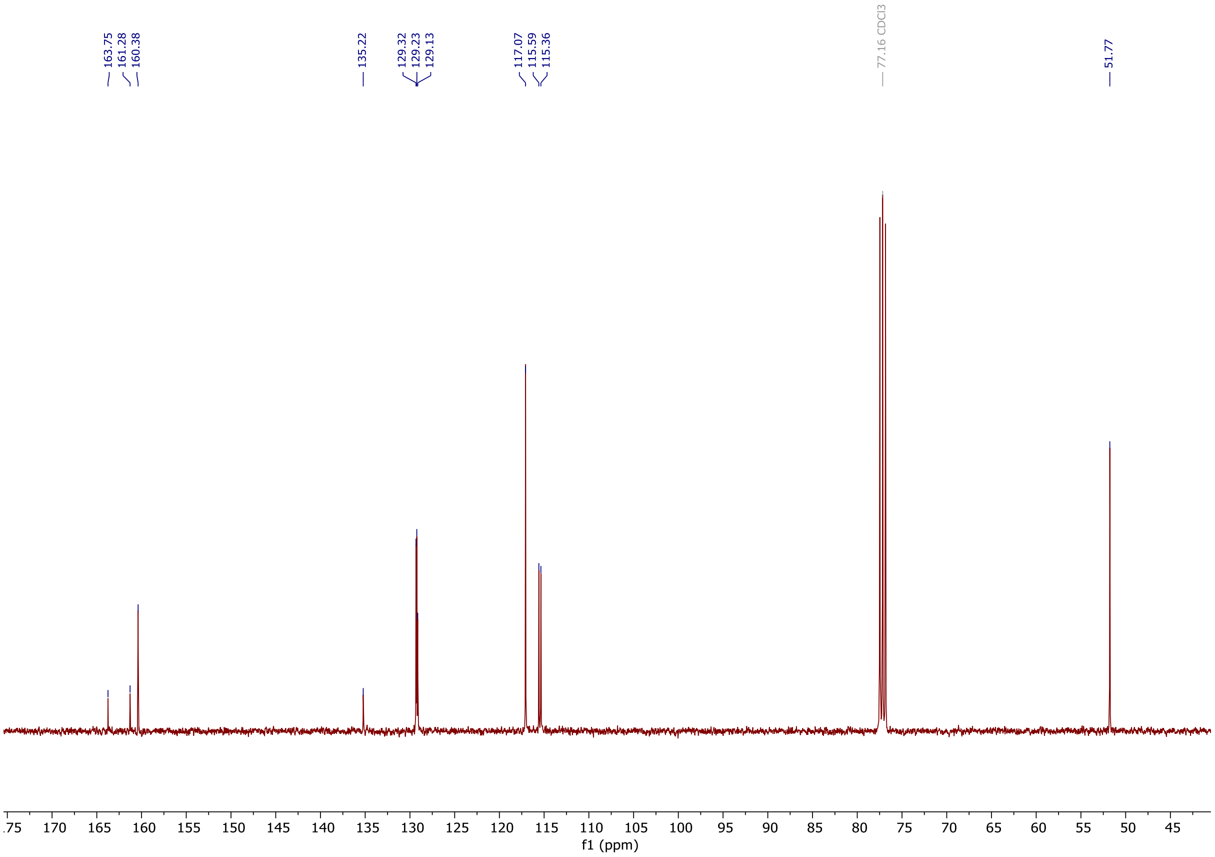
163.75
161.28
160.38
135.22
129.32
129.23
129.13
117.07
115.59
115.36
77.16 CDCl3
51.77
.75 170 165 160 155 150 145 140 135 130 125 120 115 110 105 100 95 90 85 80 75 70 65 60 55 50 45
f1 (ppm)

Dimethyl *N*-(4-cyanophenyl)pyrrole-2,5-dicarboxylate (6g)

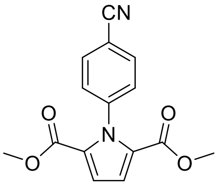

6g

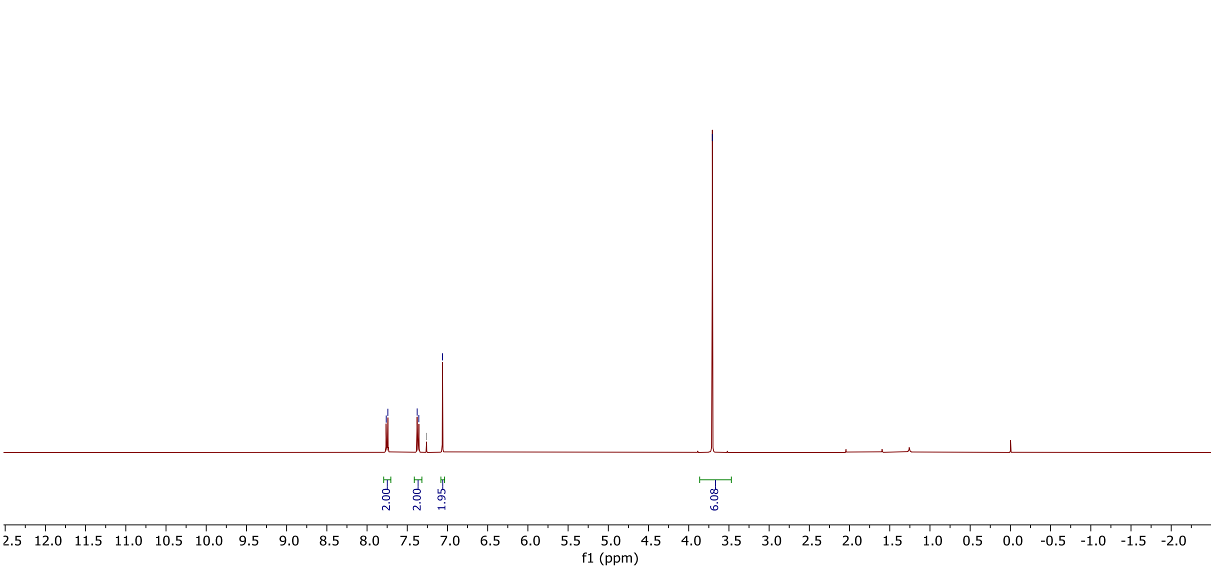

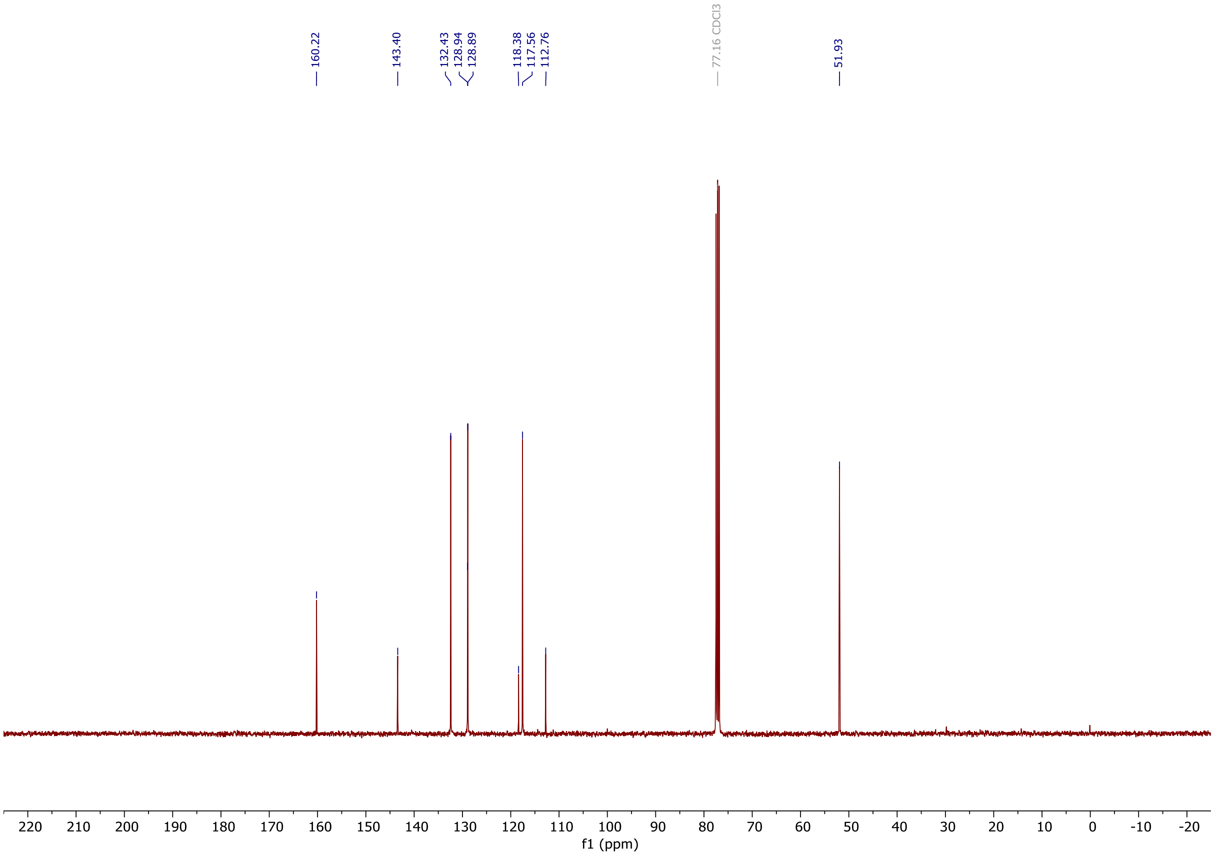

Dimethyl *N*-(4-hydroxyphenyl)pyrrole-2,5-dicarboxylate (6h)

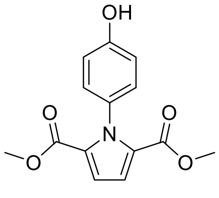

6h

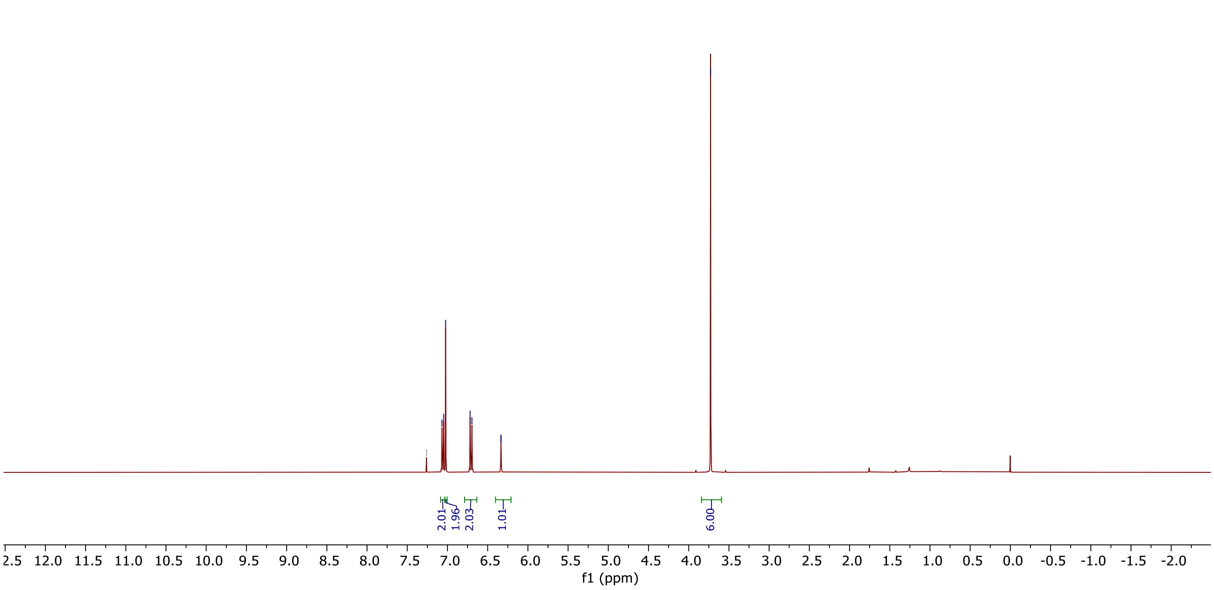

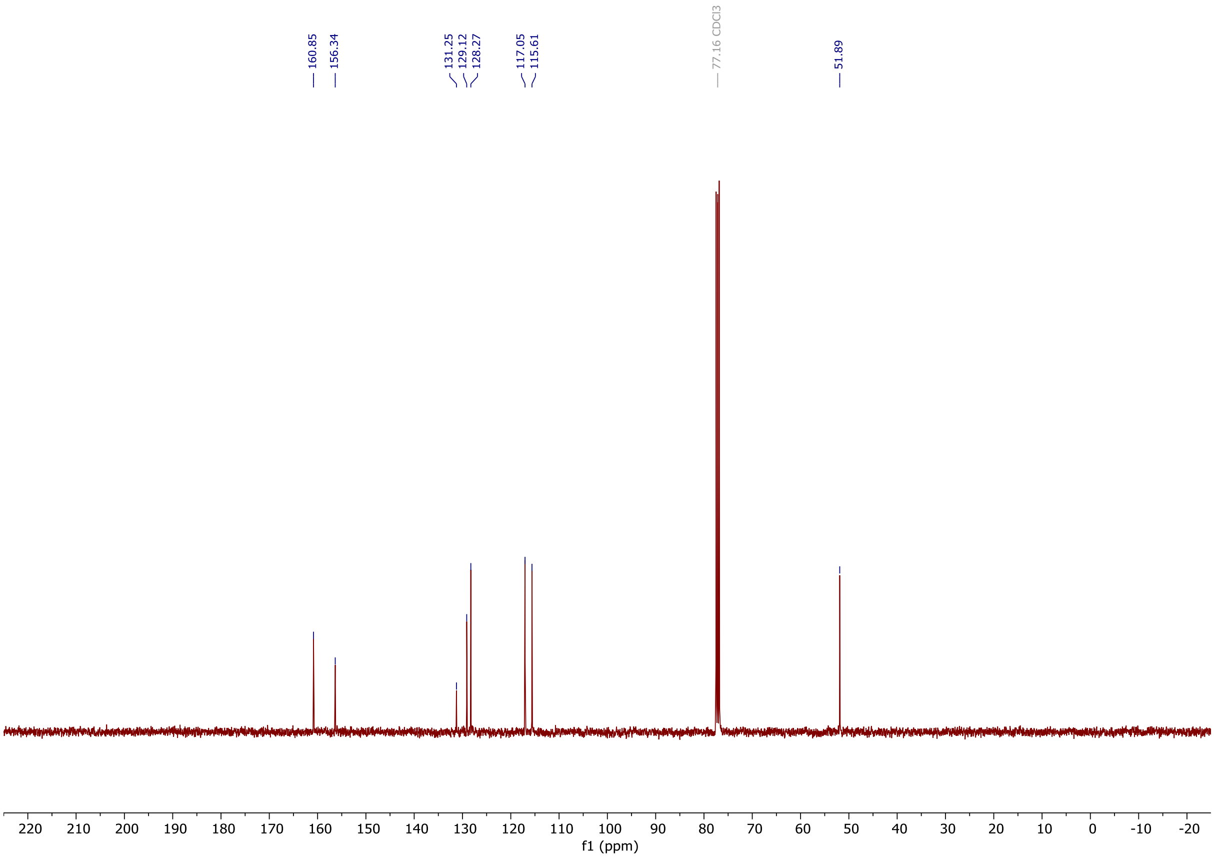

Dimethyl *N*-(4-methoxyphenyl)pyrrole-2,5-dicarboxylate (6i)

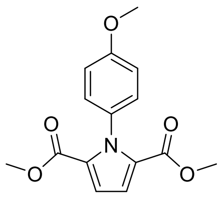

6i

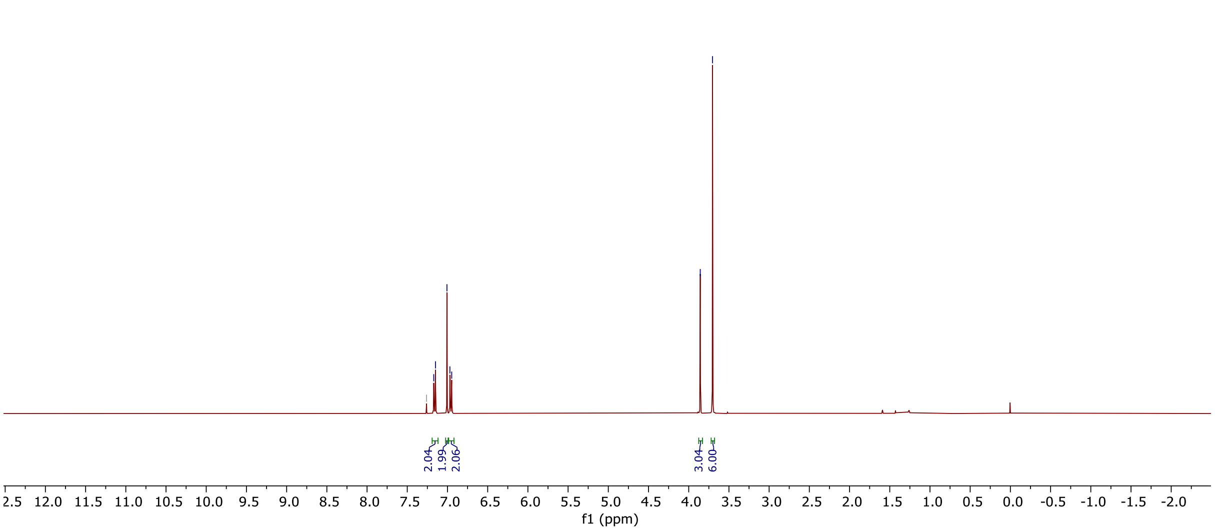

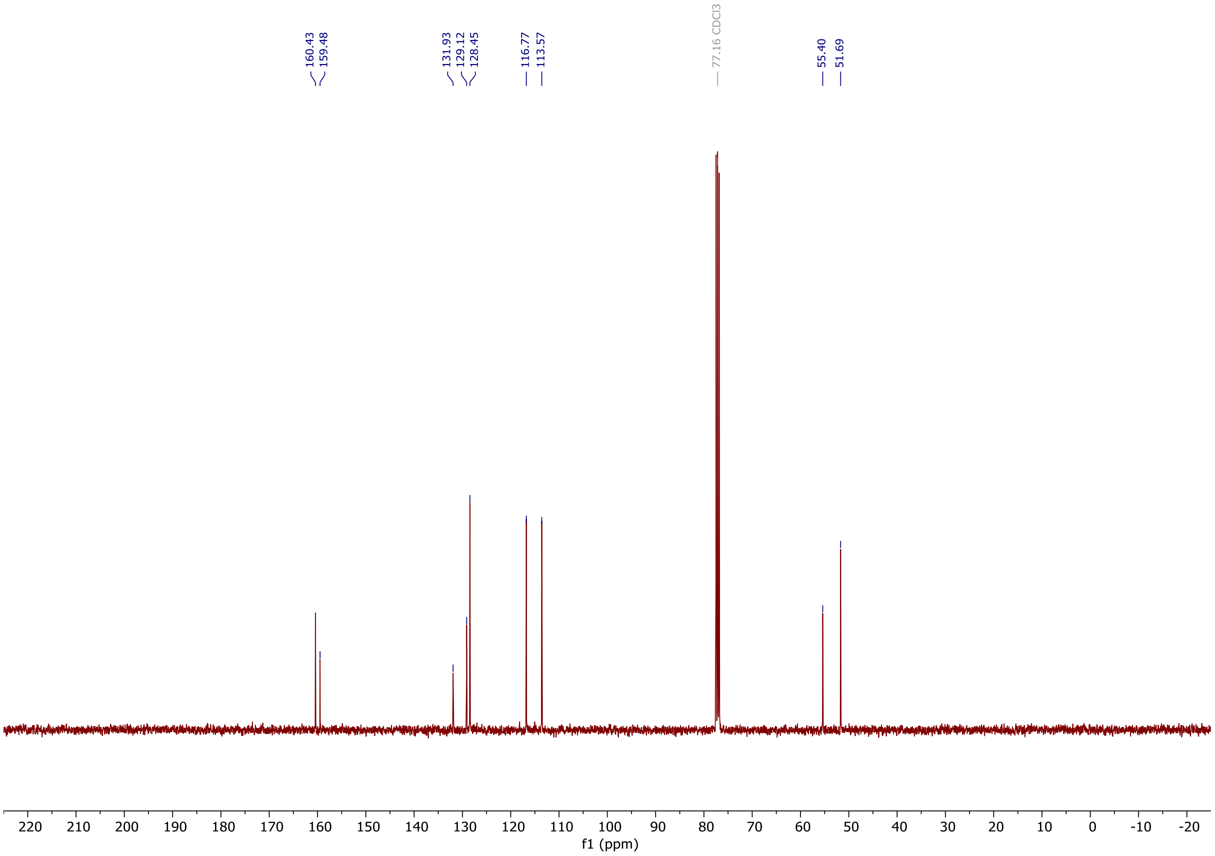

Dimethyl *N*-(4-nitrophenyl)pyrrole-2,5-dicarboxylate (6j)

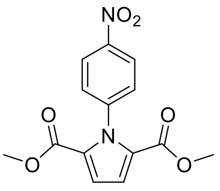

6j

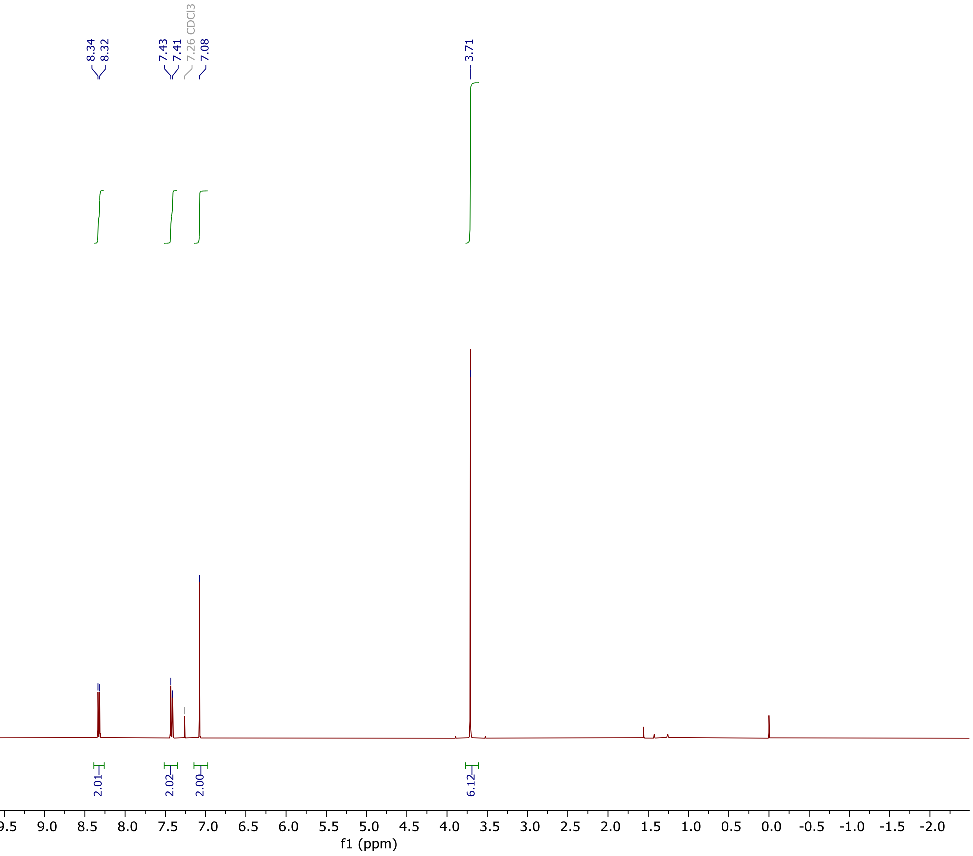

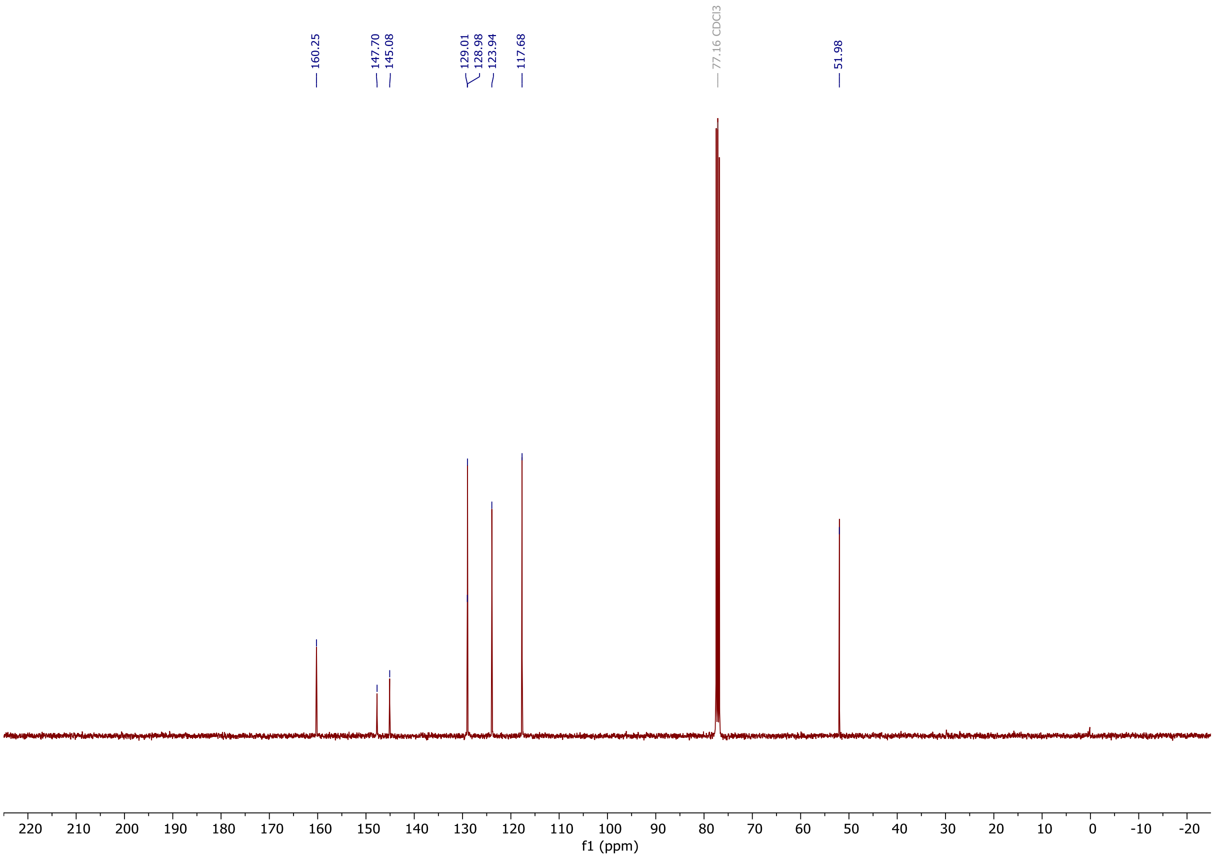

Dimethyl *N*-(2-tolyl)pyrrole-2,5-dicarboxylate (6k)

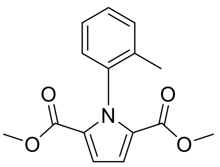

6k

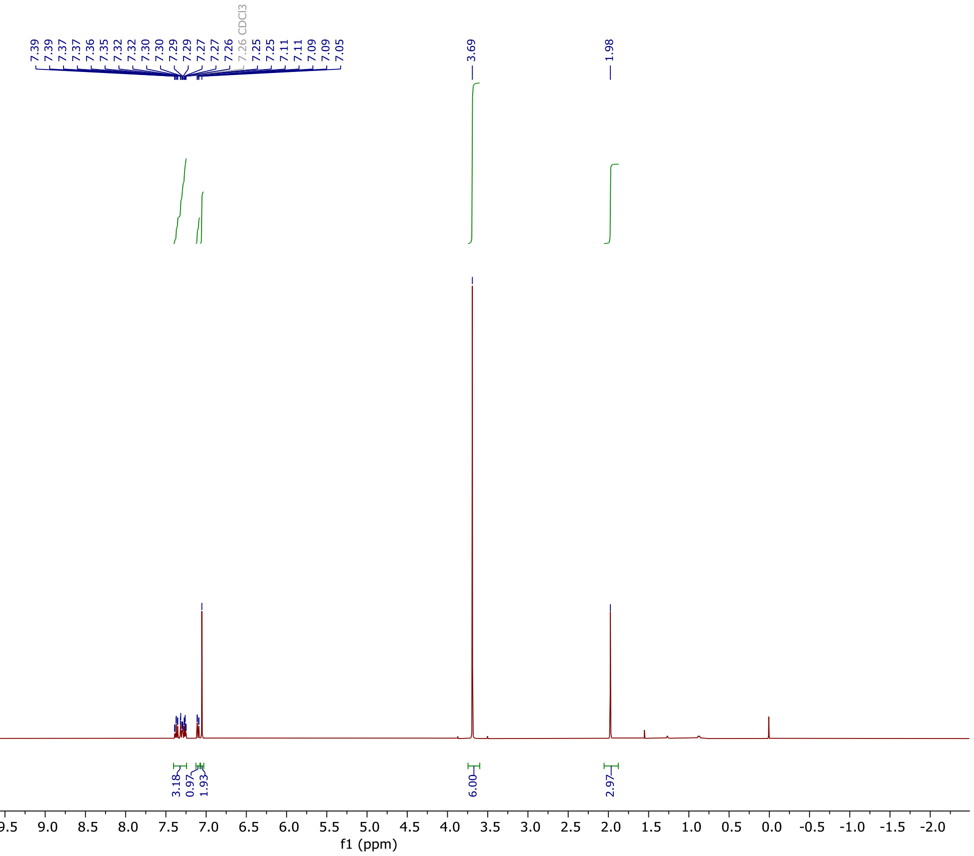

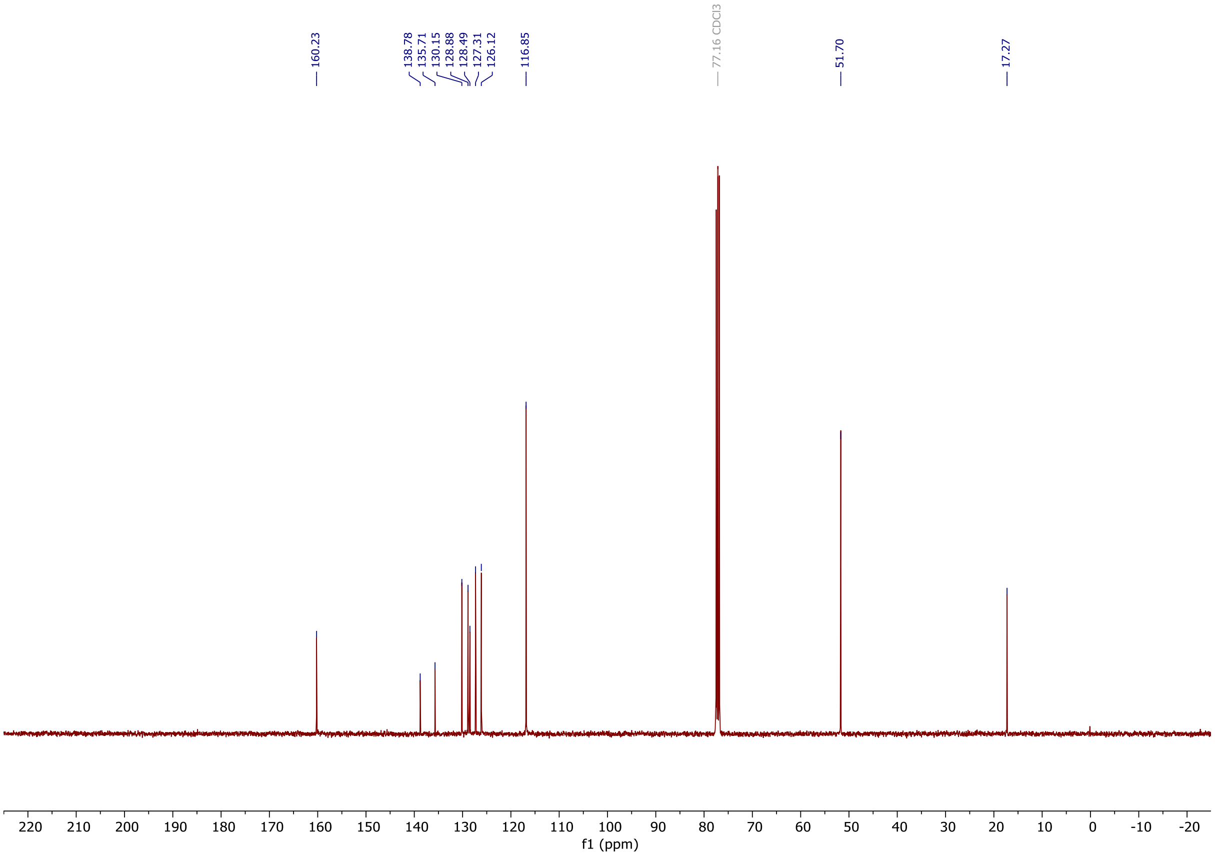

Dimethyl *N*-(2,6-dimethylphenyl)pyrrole-2,5-dicarboxylate (6l)

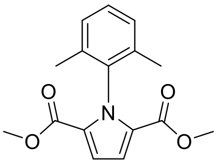

6l

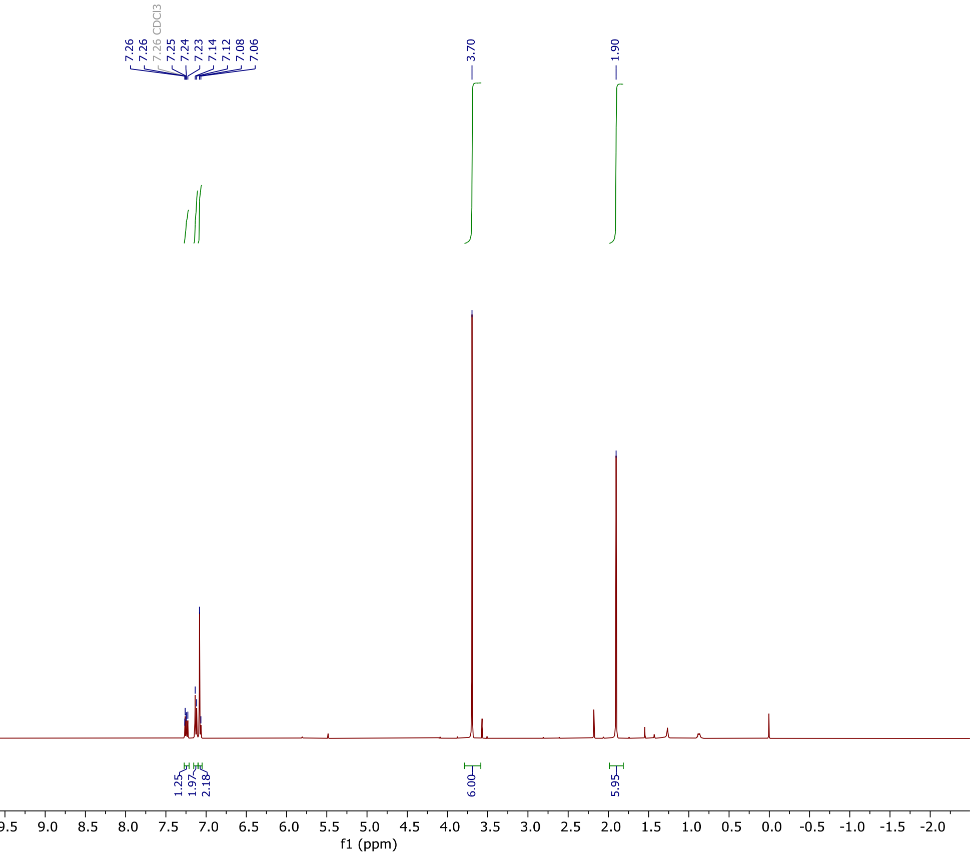

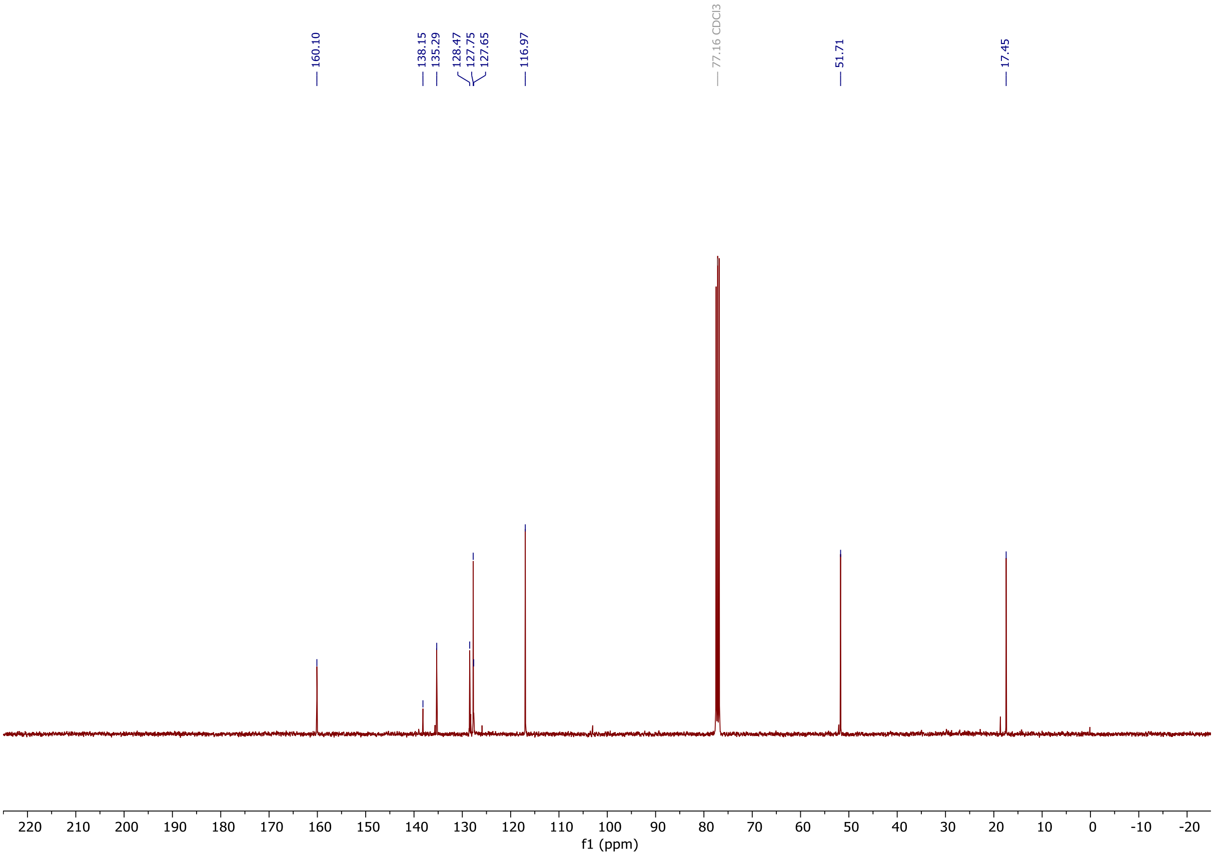

Dimethyl *N*-isopropylpyrrole-2,5-dicarboxylate (6m)

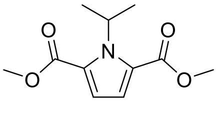

6m

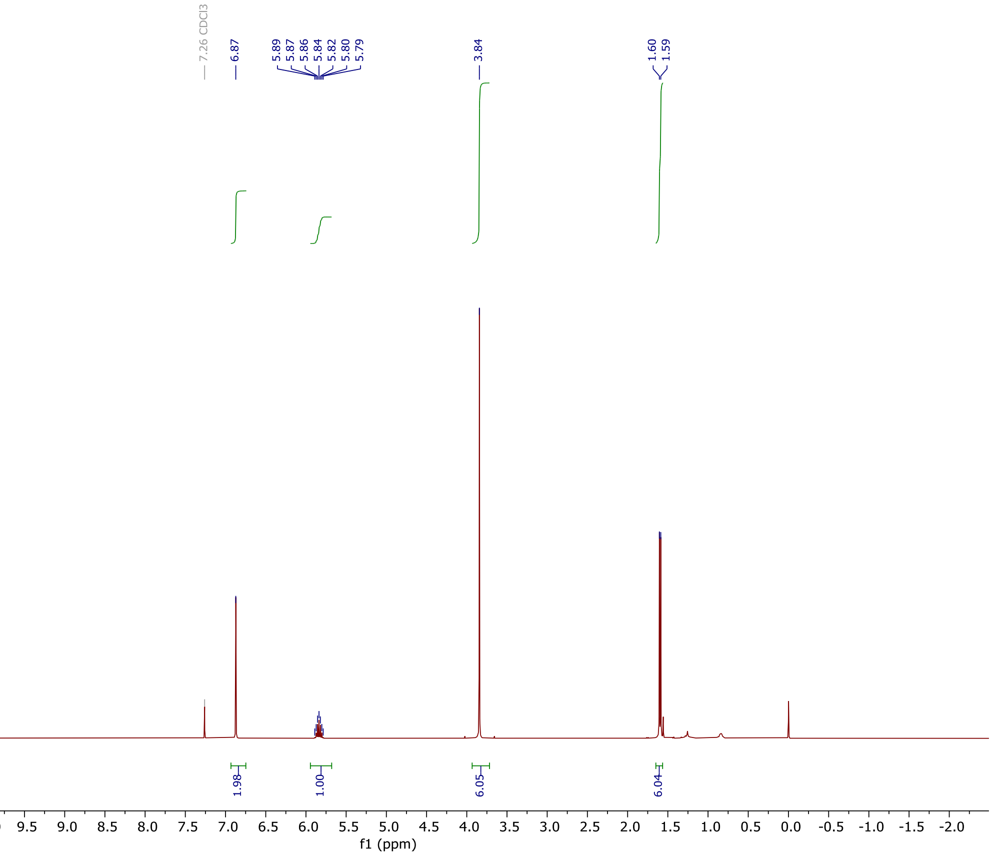

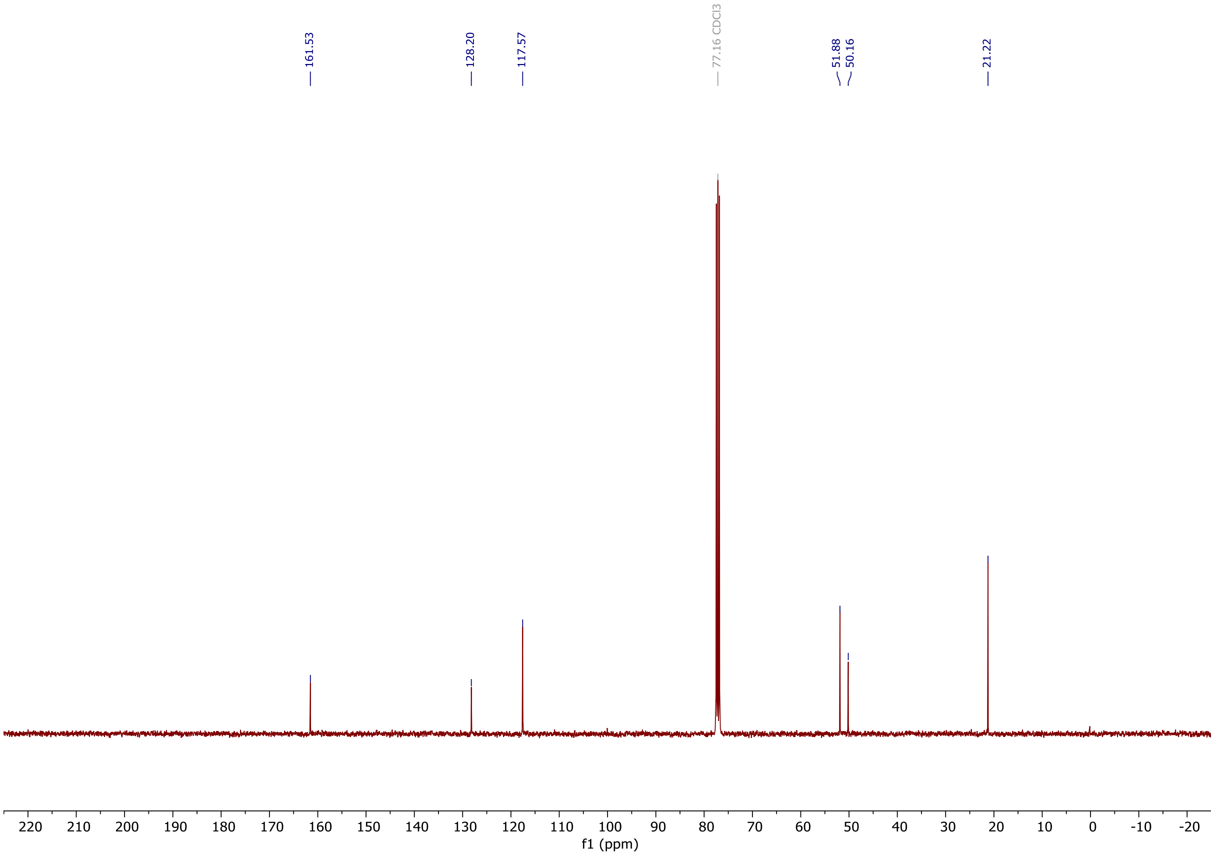

Dimethyl *N*-hexylpyrrole-2,5-dicarboxylate (6n)

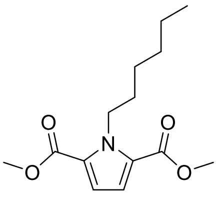

6n

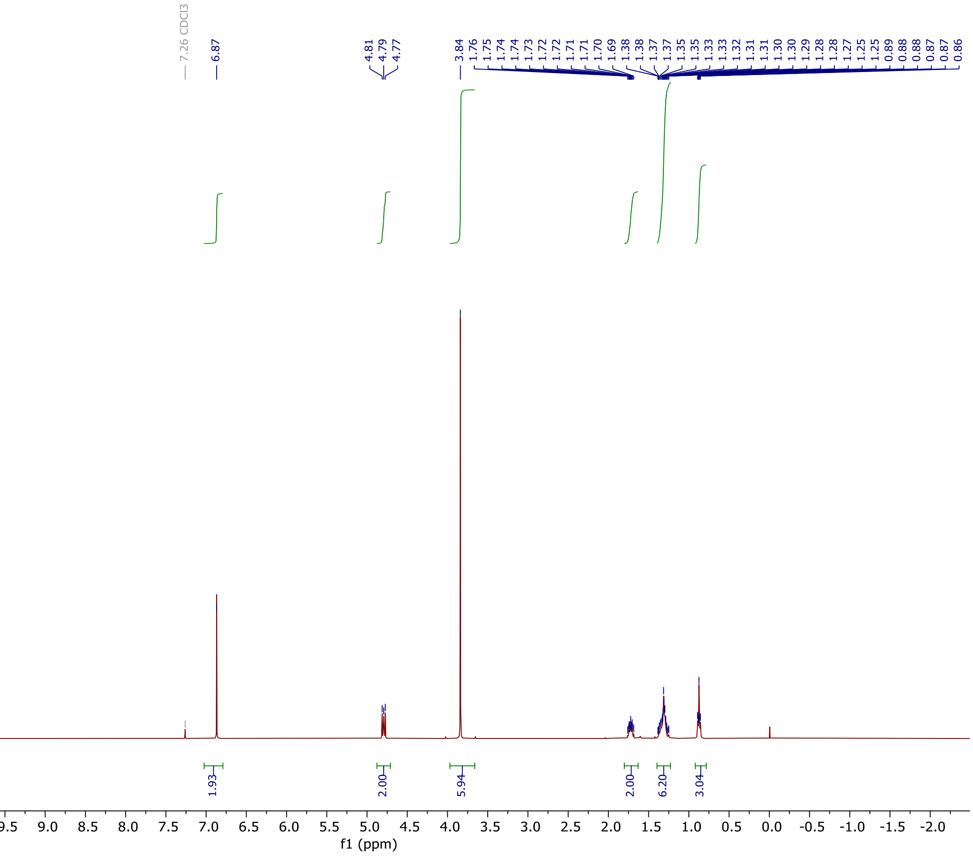

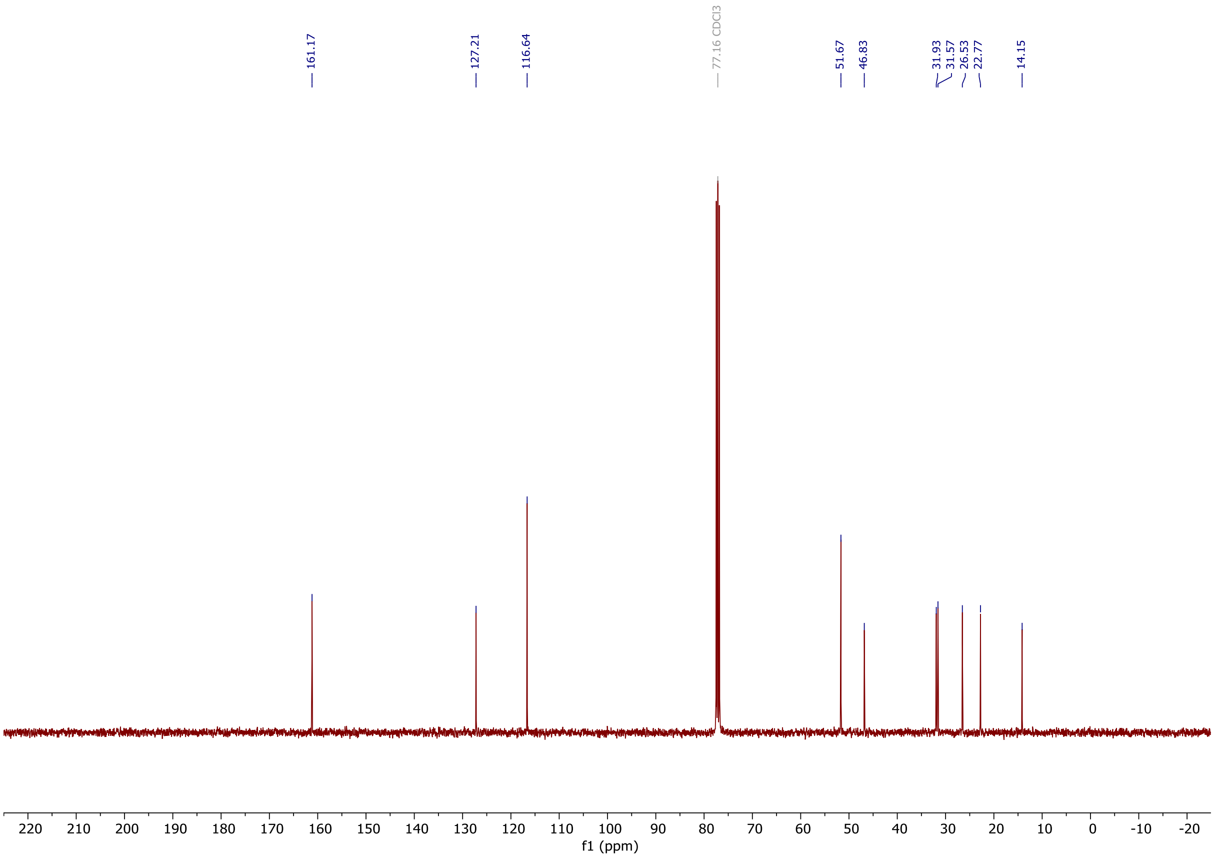

Dimethyl *N*-benzylpyrrole-2,5-dicarboxylate (6o)

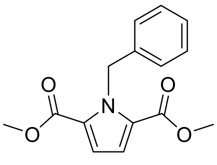

6o

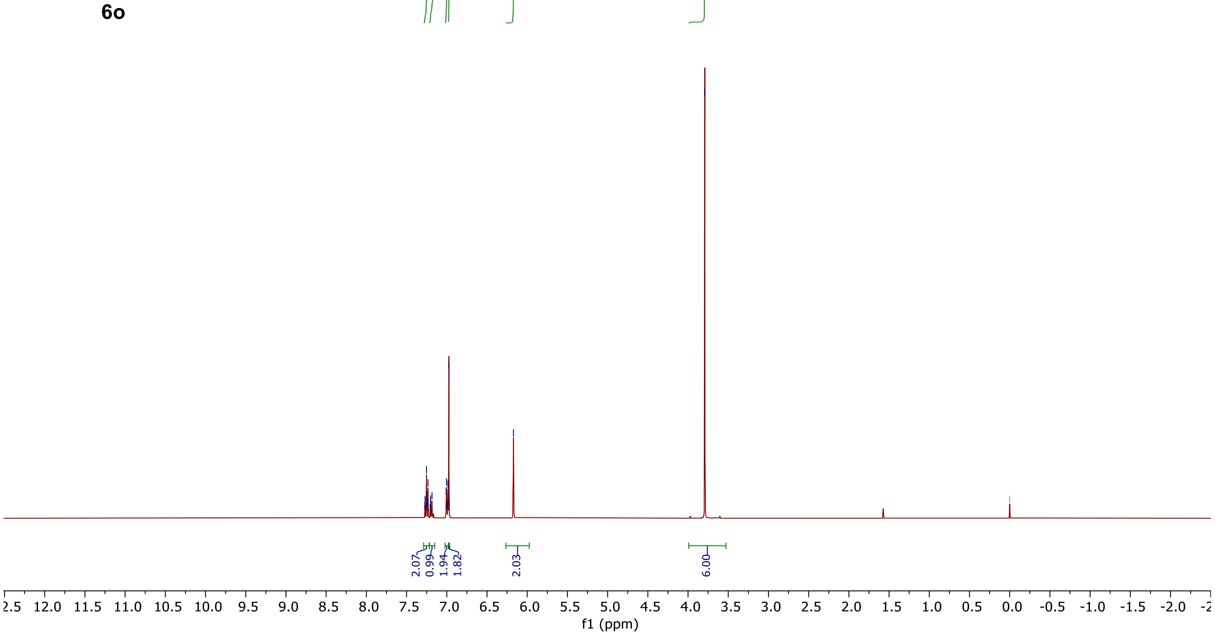

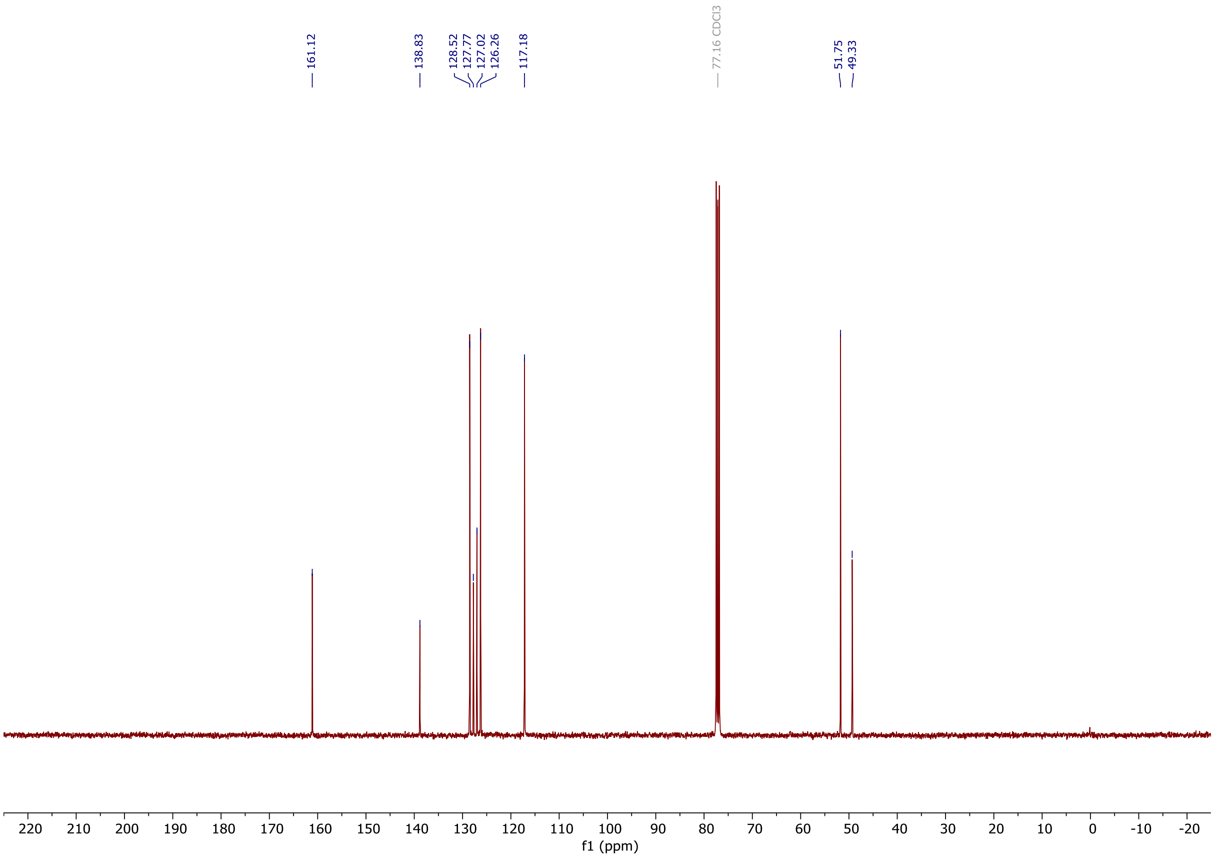

Dimethyl *N*-cycloproylpyrrole-2,5-dicarboxylate (6p)

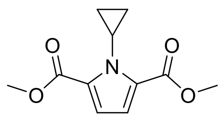

6p

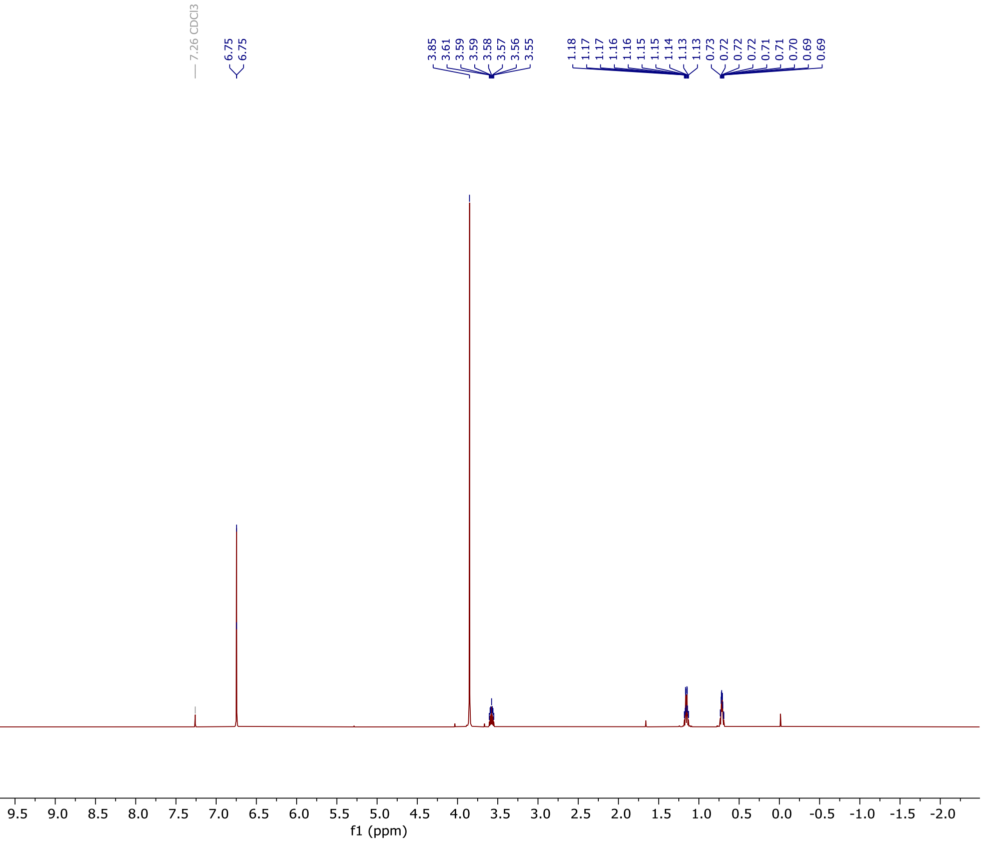

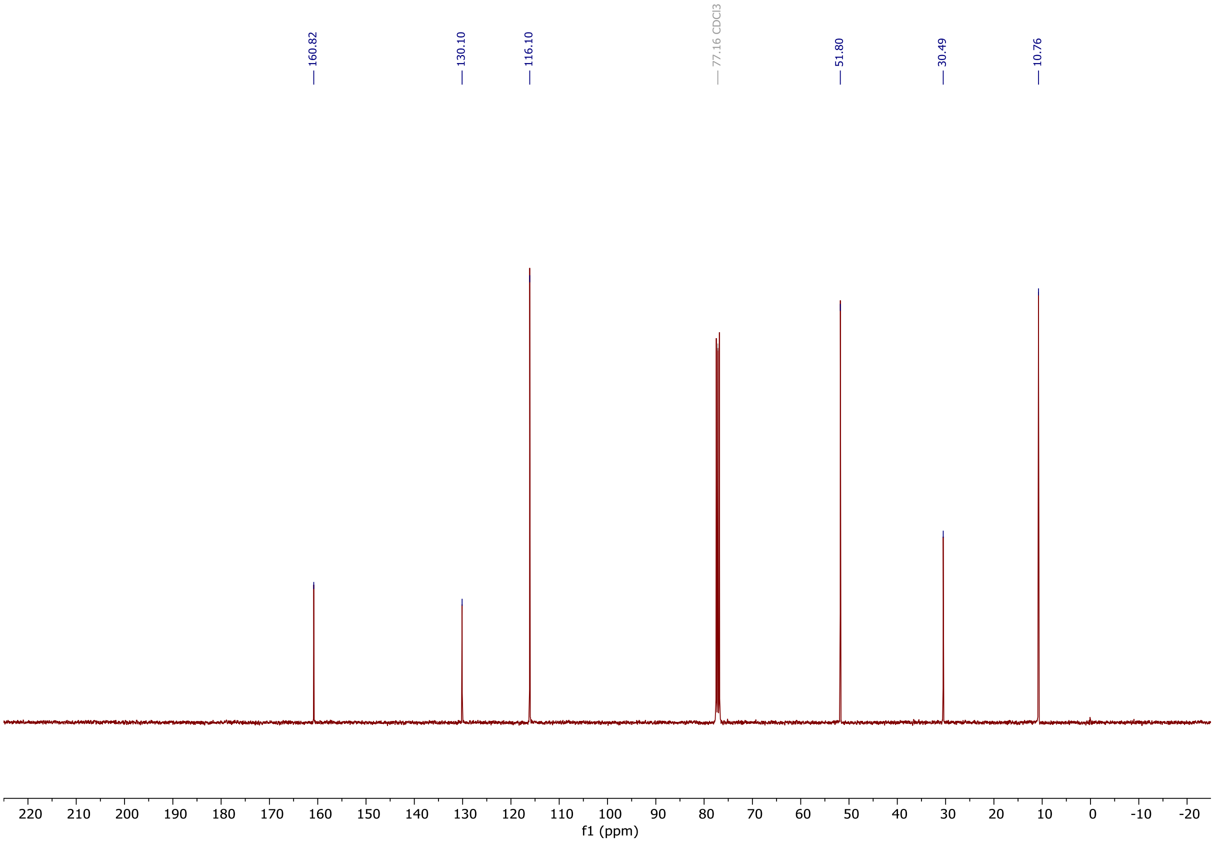

Dimethyl *N*-methylpyrrole-2,5-dicarboxylate (6q)

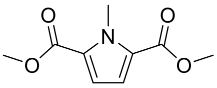

6q

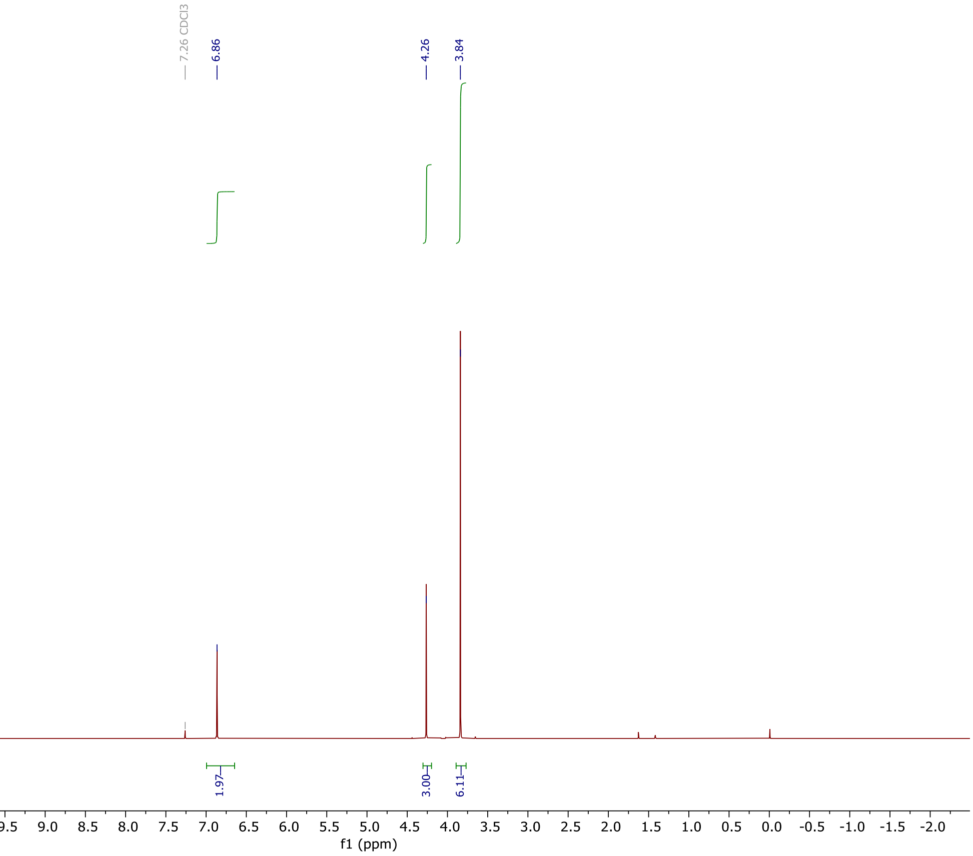

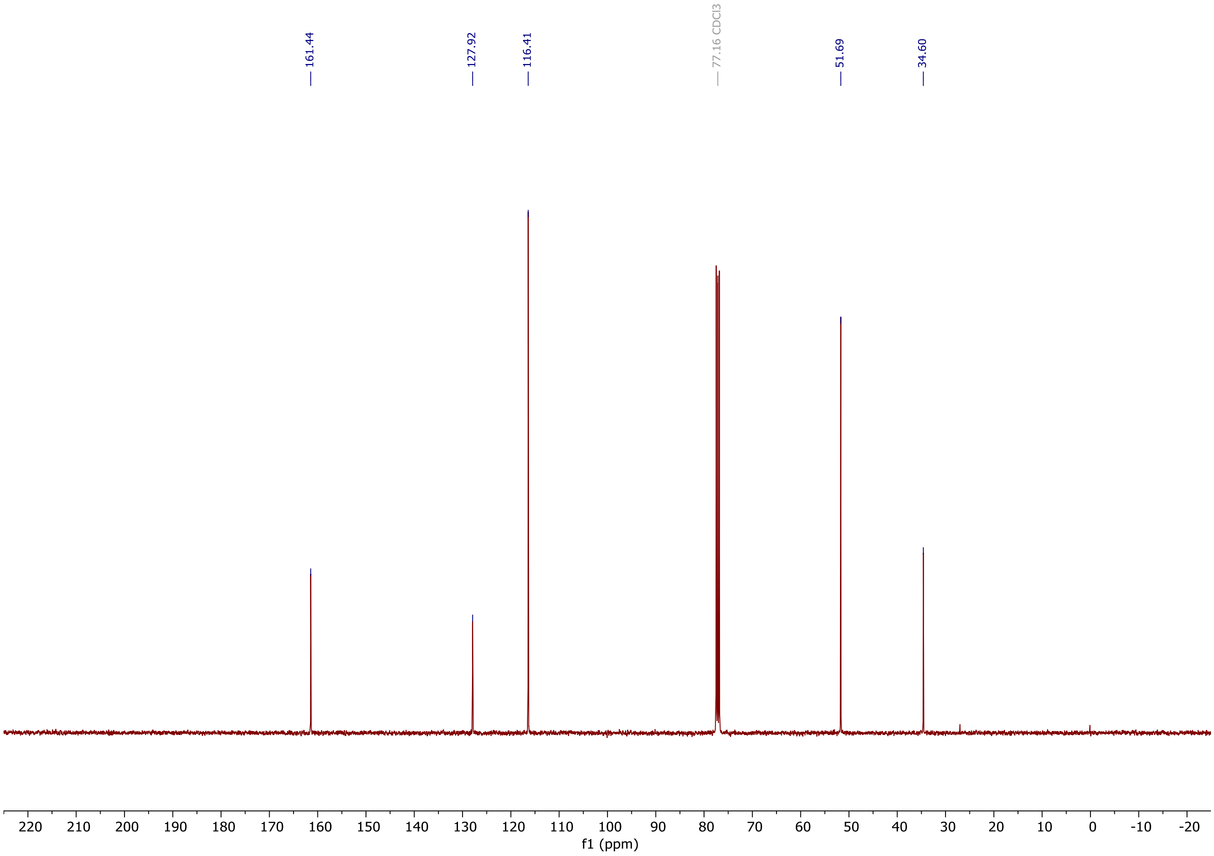

N-Phenylpyrrole-2,5-dicarboxylic acid (7a)

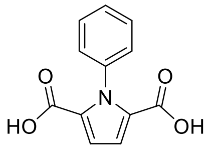

7a

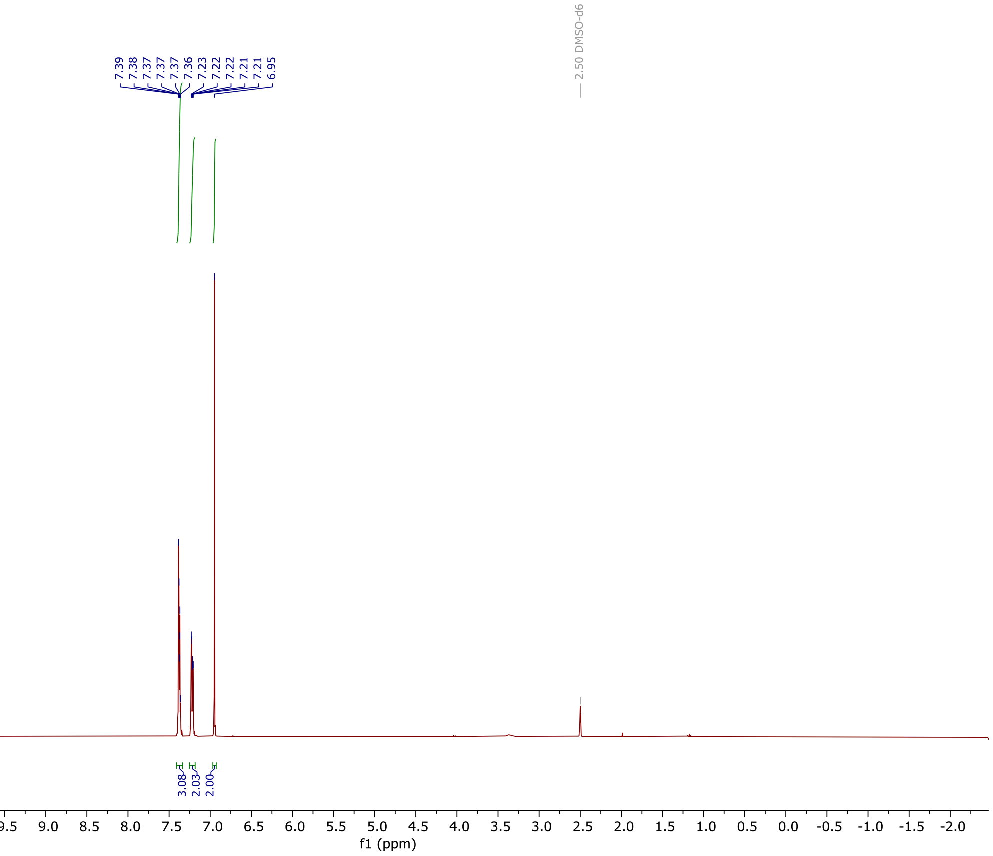

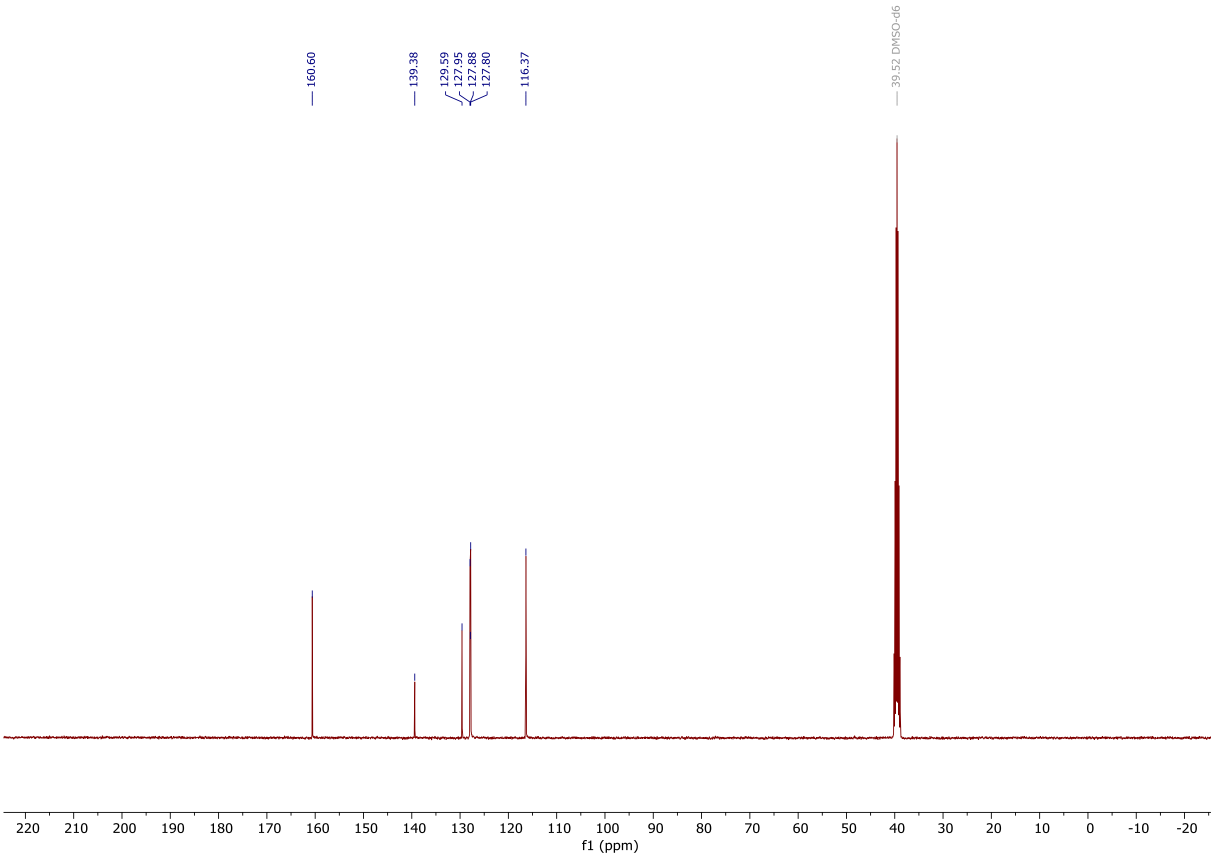

N-Benzylpyrrole-2,5-dicarboxylic acid (7b)

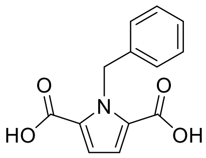

7b

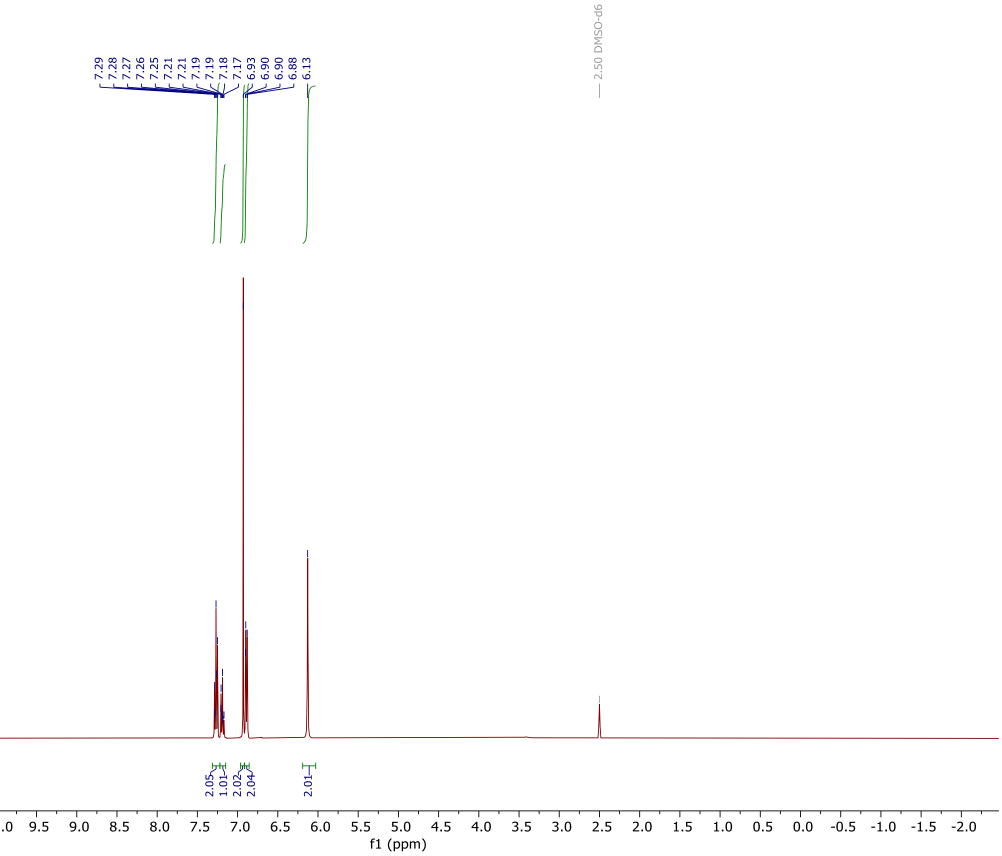

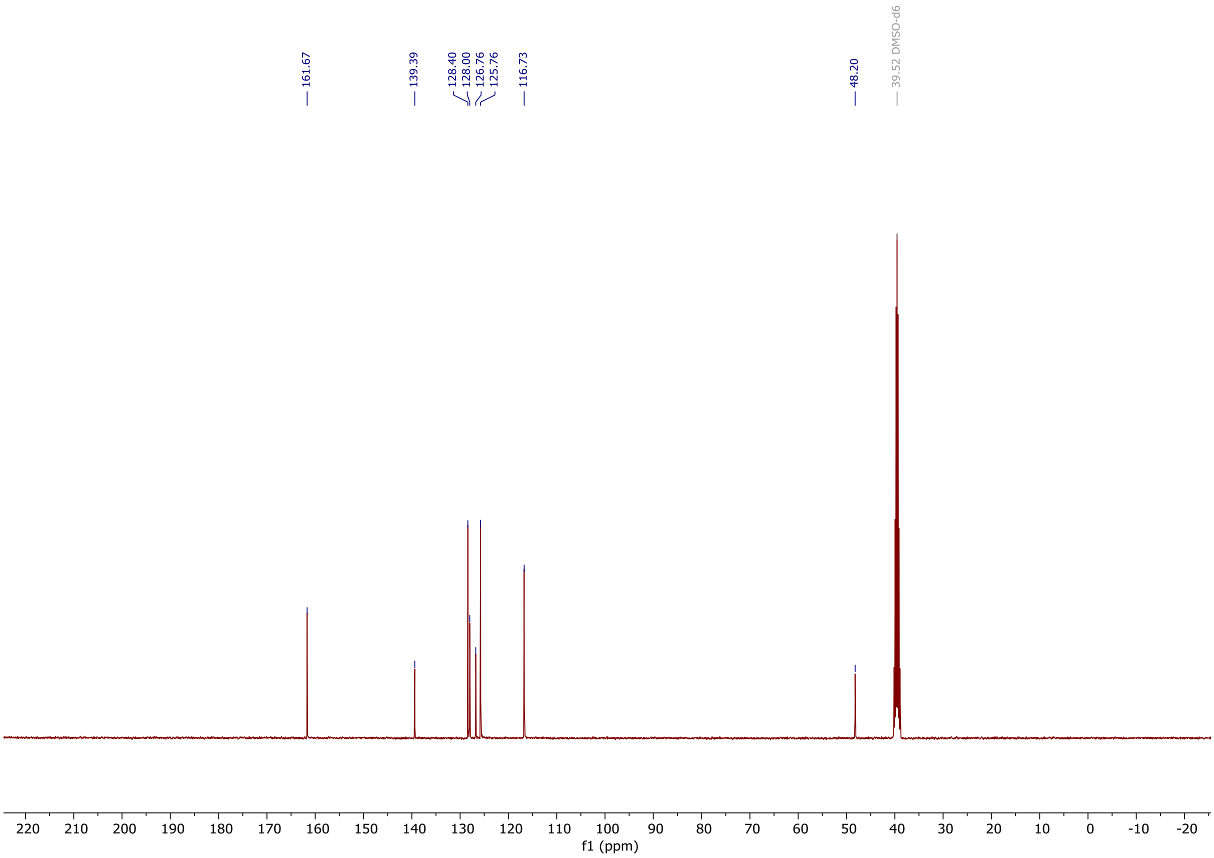

N-Methylpyrrole-2,5-dicarboxylic acid (7c)

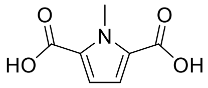

7c

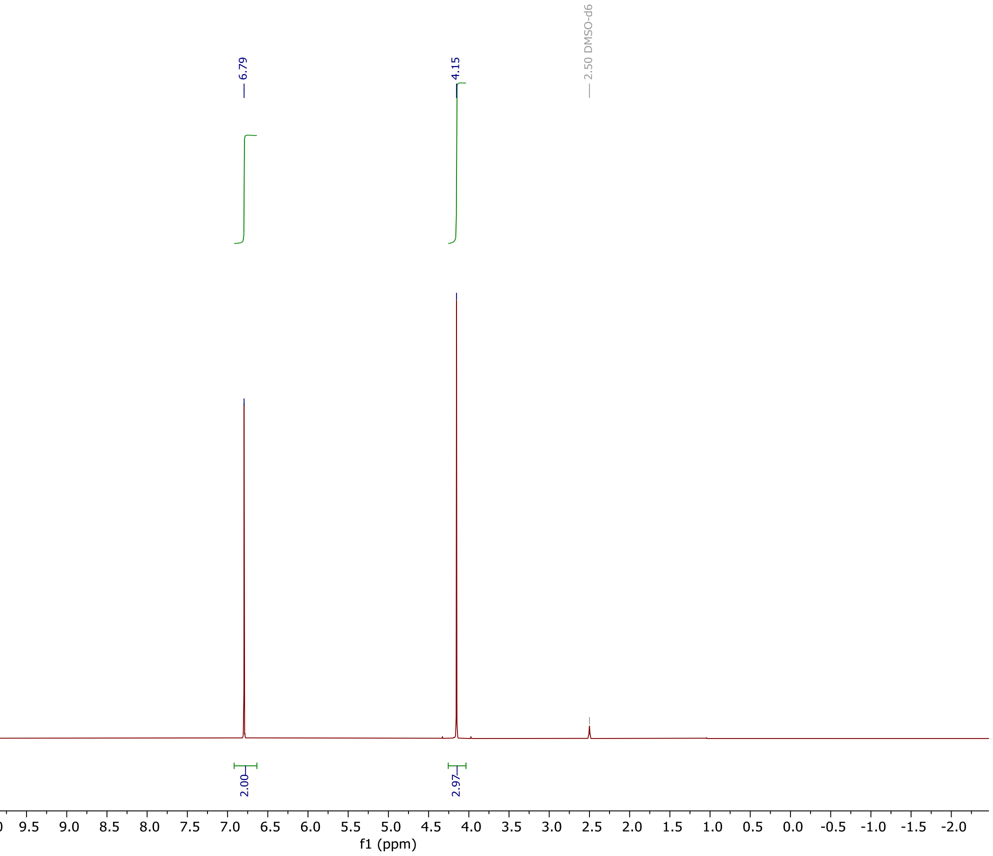

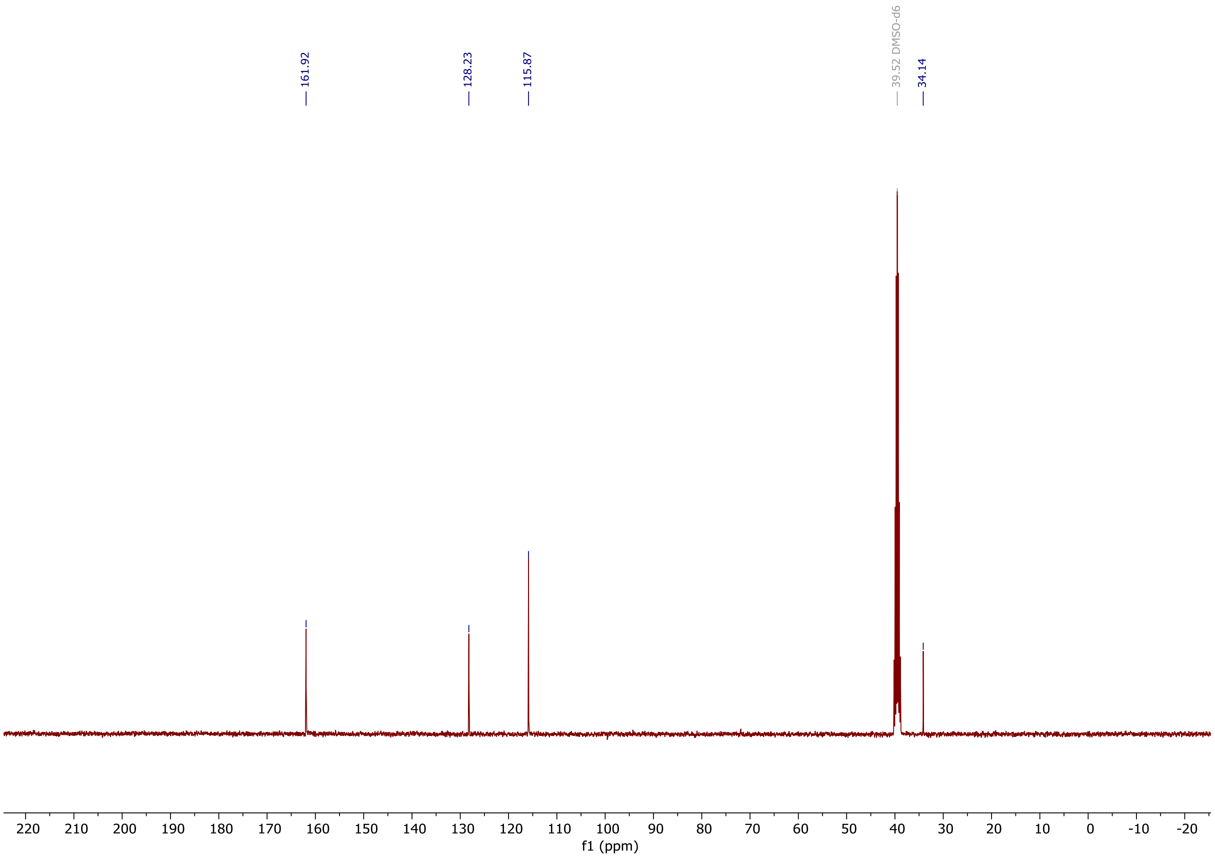

E-factor calculation

Galactaric acid route

Table 1: E-factor D-galactaric acid route

Product	molar mass [g/mol]	molar amount [mol]	mass [g]	Starting materials	solvent factor [mL/g]	mass [g]	volume [mL]	density [g/mL]	molar mass [g/mol]	molar amount [mol]	yield	equivalents	E-factor
N-phenylpyrrol-2,5-dicarboxylic acid (7a)	231,21	0,0416	9,61	Dimethyl-N-phenylpyrrole-2,5-dicarboxylic acid (6a)		11,2			259,26	0,0432			
				THF	4,46	44,4	50	0,8876					
				NaOH (2,5M)	9,82	122,1	110	1,11					17,5
Dimethyl-N-phenylpyrrole-2,5-dicarboxylic acid (6a)			11,2	Dimethyl 2,5-dihydroxymuconate (5)		8,91			202,16	0,0441	0,98	1,00	
				Aniline		4,52			93,13	0,0485		1,10	
				Saccharin		2,22			183,19	0,0121		0,25	
				MeOH	9,90	69,7	88,2	0,79					6,62
Dimethyl 2,5-dihydroxymuconate (5)			8,91	Dimethyl-2,3,4,5-tetra-O-acetylgalactarate (3)		33,8			406,34	0,0832	0,53	1,00	
				DBU		27,1			152,24	0,1780		2,14	
				Chloroform	2,46	123	83,1	1,48					
				Acetyl chloride		4,90			78,5	0,0624		0,75	
				MeOH	3,50	93,4	118	0,79					30,7
Dimethyl-2,3,4,5-tetra-O-acetylgalactarate (3)			33,8	Dimethylgalactarate (2)		21,8			238,19	0,0914	0,91	1,00	
				Sulfuric acid		2,00	1,09	1,84					
				Acetic anhydride	5,00	118	109	1,08					3,18
Dimethylgalactarate			21,8	Galactaric acid		19,6			210,14	0,0933	0,98	1,00	
				Sulfuric acid		1,80	0,98	1,84					
				Methanol	7,00	108	137	0,79					4,95
												**E-factor (total)**	62,9

Ethyl bromopyruvate route

Table 2: E-factor ethyl bromopyruvate route.

Product	molar mass [g/mol]	molar amount [mol]	mass [g]	Starting materials	solvent factor [mL/g]	mass [g]	volume [mL]	density [g/mL]	molar mass [g/mol]	molar amount [mol]	yield	equivalents	E-factor
N-phenylpyrrol-2,5-dicarboxylic acid	231,21	0,0416	9,61	Diethyl N-phenylpyrrol-2,5-dicarboxylate		15,7			287,32	0,0547	0,76	1,00	
				KOH		53,7			56,105	0,957		17,5	
				Ethanol	500	6202	7857	0,78945					652
Diethyl N-phenylpyrrol-2,5-dicarboxylate			15,7	Diethyl-2,5-dihydroxymuconate		19,7			230,22	0,0855	0,64	1,00	
				Aniline		15,9			93,13	0,171		2,00	
				Acetic acid	137	2830	2695	1,05					
													182
Diethyl-2,5-dihydroxymuconate			19,7	Ethyl bromopyruvat		130			167,00	0,777	0,11	1,00	
				Zinc		25,4			65,38	0,388		0,50	
				Acetone	4,75	487	616	0,79					31,6
												**E-factor (total)**	865

$$E\ factor = \frac{\sum m\ (raw\ materials) + \sum m(reagents) + \sum m(solvents) - m(desired\ product)}{m\ (desired\ product)} \quad (1)¶$$

Literature

[1] J.-S. Jeshua Friedrichs, L. Schmermund, C. Urmann, V. Sieber, *Helv. Chim. Acta* **2024**, *107*, e202400036.

[2] Q. Lin, S. J. Geib, A. D. Hamilton, *J. Chem. Soc., Perkin Trans. 2* **1998**, 2109-2116.
